# Sex and educational inequalities across functional ability trajectories in Chile: A sequence analysis of a 17-year national panel

**DOI:** 10.1371/journal.pone.0361826

**Published:** 2026-11-03

**Authors:** Ignacio Cabib, Pablo Villalobos Dintrans, Jorge Browne Salas

**Affiliations:** 1 Instituto de Sociología & Escuela de Salud Pública, Pontificia Universidad Católica de Chile, Santiago, Chile; 2 Centro UC Estudios de Vejez y Envejecimiento, Santiago, Chile; 3 Facultad de Medicina y Ciencias de la Salud, Universidad Mayor, Santiago, Chile; 4 Centro de Observación y Análisis de Datos en Salud (CADS), Universidad Mayor, Santiago, Chile; 5 Millennium Institute for Care Research (MICARE), Santiago, Chile; 6 Sección de Geriatría, Facultad de Medicina, Pontificia Universidad Católica de Chile, Santiago, Chile; Menzies School of Health Research: Charles Darwin University, AUSTRALIA

## Abstract

**Context:**

Research on disability and functional ability has increasingly moved from cross-sectional descriptions toward longitudinal analyses of intra-individual change. This shift has shown that functional ability follows heterogeneous pathways, encompassing stability, progressive deterioration, fluctuating limitations, partial recovery, and death. However, evidence remains concentrated in high-income countries, and fewer studies have examined long-term functional ability trajectories in Latin American contexts marked by rapid population aging and high socioeconomic inequality. In addition, comparisons across sex and educational groups require trajectory typologies that are constructed in a way that makes group comparisons methodologically comparable.

**Methods:**

We used six observation points from the Chilean Social Protection Survey, a nationally representative longitudinal survey, covering the period 2004–2020. The analytic sample included 2,681 individuals aged 46–65 at baseline, contributing 16,086 person-wave observations. Functional ability was measured through self-reported limitations in activities of daily living and classified into five states: no limitation, low limitation, middle limitation, high limitation, and deceased. We reconstructed individual functional ability sequences over 17 years and applied optimal matching followed by hierarchical clustering. To ensure comparability across sex and educational groups, trajectory typologies were estimated within each baseline age group using the full age-group-specific sample. We then examined how membership in these pooled trajectory types varied by sex and educational attainment, referencing the composition of each trajectory type to the overall composition of its baseline age group.

**Findings:**

Across the four baseline age groups, detailed trajectories were grouped into three broader types: mostly non-declining, progressive-declining, and equivocal-declining. Mostly non-declining trajectories were prevalent in all age groups, although their internal composition changed with baseline age, moving from stable good functional ability in younger age groups toward preserved functional ability before death in older age groups. Progressive-declining trajectories were especially visible among respondents aged 56–60 at baseline and remained substantial among those aged 61–65. Equivocal-declining trajectories were more frequently composed of women, most markedly among respondents aged 61–65 at baseline, and of respondents with primary education or less in the three younger age groups. Sex differences in trajectory membership were statistically significant in all age groups, while educational differences were statistically significant in the first three age groups.

**Conclusions:**

Long-term functional ability trajectories in Chile are heterogeneous and socially patterned. By deriving pooled trajectory typologies within each baseline age group before comparing sex and educational groups, and by referencing group composition to the marginal composition of each age group, this study provides comparable evidence on how functional aging unfolds across socially stratified groups in an understudied Latin American context.

## Introduction

Functional ability research has changed substantially over recent decades. Earlier approaches often examined disability or functional limitations as static outcomes observed at one point in time. More recent research has emphasized the dynamic nature of functional ability, conceptualizing it as a process that unfolds through transitions between independence, limitation, recovery, worsening health, and death. This shift has been supported by the availability of longitudinal data and by methodological approaches capable of representing individual trajectories over extended periods. The concept of functional ability has also become increasingly relevant because it moves the analytical focus from disability as a fixed attribute toward people’s capacities and limitations in daily life, consistent with broader changes in the terminology and conceptualization of disability and aging [1,2].

A central contribution of longitudinal disability and functional ability research has been the identification of heterogeneous trajectories. The importance of examining disability as a dynamic process rather than as a single status measured at one point in time has been increasingly emphasized [2,3]. Research has shown that functional aging follows several distinct pathways. Some individuals maintain high functional ability over long periods, others experience gradual deterioration, and others move between lower and higher levels of limitation. Later-life health has been characterized as involving gradual change, homeostasis, and punctuated equilibrium, which draws attention to stability, instability, and non-linear transitions [3]. Previous research in Chile has also shown that functional ability trajectories include stable, progressive-declining, equivocal-declining, and mortality-related patterns [4].

This trajectory-based perspective has also raised questions about the social patterning of functional ability, and evidence from high-income settings has begun to specify the channels through which such patterning operates. Sex differences are consistently documented, with women accumulating more functional limitations and more unstable patterns while surviving longer, a configuration linked to a higher burden of non-fatal disabling conditions, to differences in muscle mass and strength, and to lifetime differences in paid work and caregiving responsibilities [5–8].

Socioeconomic position operates through a broader set of intermediate mechanisms. Educational attainment shapes exposure to physically demanding and hazardous occupations, health literacy and the capacity to navigate health systems, income and material conditions in later life, and health behaviors such as physical activity [9–13]. Chronic conditions, multimorbidity, and sedentary behavior operate both as precipitating events that trigger transitions into limitation and as constraints on the likelihood of regaining function once limitation has occurred, so that advantaged groups more frequently display recovery and less frequently remain in unstable states [14–18].

These mechanisms accumulate across the life course, with early-life socioeconomic circumstances and adulthood employment histories shaping functional status decades later [19]. Evidence from Taiwan, China, Hong Kong, the United States, Japan, and the United Kingdom converges in showing that functional decline is shaped by this configuration of individual and social factors rather than by chronological age alone, although the strength and even the direction of specific associations varies across age groups and cohorts [7,8,12–18].

## Research gaps

Despite these advances, two gaps remain relevant. First, most evidence on long-term functional ability trajectories comes from the United States, Europe, and parts of Asia [7,8,12–18]. Evidence from Latin America remains more limited, even though the region is characterized by rapid population aging, high socioeconomic inequality, and institutional arrangements that differ from those observed in countries where most trajectory studies have been conducted. Chile is an especially relevant setting because it combines rapid demographic aging, pronounced social inequalities, and a segmented health and social protection system.

These features carry specific implications for how functional ability trajectories are expected to unfold. Demographic aging in Chile is occurring at a pace that compresses into a few decades a transition that took considerably longer in the settings where most trajectory evidence has been produced, so that successive cohorts reach later life with markedly different educational and occupational endowments. Educational expansion has been rapid, and the proportion of individuals with primary education or less rises steeply across the cohorts examined here, which suggests that educational differences in functional trajectories will be entangled with cohort composition and that the social meaning of a given educational threshold shifts across baseline age groups [20].

The Chilean labor market has been characterized by weak employment protection and persistent informality, conditions that concentrate cumulative exposure to physically demanding and unprotected work among lower-educated individuals and that plausibly produce steeper educational gradients than those observed in settings with stronger labor regulation [19]. The health system is segmented by ability to pay, and long-term care provision remains limited, with families absorbing most of the care burden, which makes the likelihood of regaining function after an episode of limitation dependent on household resources and suggests that socioeconomic differences will be expressed in the stability and reversibility of trajectories as much as in their onset [4,21].

Alongside these context-specific expectations, the basic heterogeneity of functional pathways, encompassing sustained stability, ordered decline, fluctuation, and mortality-related patterns, is expected to resemble that documented elsewhere, since it reflects demographic and biological regularities that are common across settings. Previous studies in Chile have extended functional ability trajectory analysis to this national context, [4] and have examined the association between long-term functional ability trajectories and mental health during the COVID-19 period [22]. However, evidence remains limited on how sex and educational inequalities are distributed across comparable long-term functional ability trajectories.

Second, the methodological basis for comparing trajectories across social groups requires careful attention. If long-term trajectories are estimated separately within sex or educational groups, the resulting clusters may represent different empirical constructs. Under that design, a trajectory labeled as stable, declining, or fluctuating in one group may not be directly equivalent to a trajectory with the same label in another group. Comparative analyses therefore require a common trajectory typology constructed before examining group differences. This approach allows sex and education to be analyzed as dimensions associated with membership in shared trajectory categories rather than as criteria used to create separate and potentially non-equivalent cluster solutions.

The present study extends previous Chilean research on functional ability trajectories in three ways. First, it uses a 17-year observation window to examine functional ability from midlife into older age, building on prior work using the Chilean Social Protection Survey [4,22]. Second, it estimates pooled trajectory typologies within baseline age groups, allowing sex and educational differences to be examined across comparable trajectory categories. Third, it groups detailed trajectories into three broader types (mostly non-declining, progressive-declining, and equivocal-declining), to describe the main ways in which functional ability changes across adulthood and older age in Chile.

The study asks: How are sex and educational attainment associated with membership in long-term functional ability trajectories among Chilean adults observed from midlife into older age? By addressing this question, the article contributes to life-course and aging research by extending trajectory-based evidence to an understudied Latin American context and by clarifying how sex and educational inequalities are expressed across comparable functional ability pathways.

## Materials and methods

### Data source and study design

We used data from the *Encuesta de Protección Social* (Social Protection Survey, EPS), a nationally representative Chilean longitudinal survey [23]. The EPS provides publicly available microdata for research on social protection, employment histories, education, income, health, household characteristics, and related dimensions.

The EPS has been fielded in 2002, 2004, 2006, 2009, 2012, 2015, 2018, 2020, and 2024. The present study used six observation points with functional ability information covering the period 2004–2020. The 2002 wave was excluded because questions on functional ability status were incorporated into the survey only from the 2004 wave onwards, and because its sample was restricted to individuals affiliated to the pension system, which limits its representativeness of the adult population [23]. The 2012 wave was published with reservations by the institution responsible for the survey, owing to low response rates during fieldwork and an accompanying recommendation that these data be used with caution for statistical inference, and it was therefore excluded from the analytic window [23]. The 2024 wave had not been released at the time the analyses were conducted and will be incorporated into future extensions of this work. This observation window provides an extended observation period in which functional ability changes can be observed from midlife into older age. The length of the panel is especially relevant for identifying different types of trajectories, including stable functional ability, gradual decline, fluctuating limitation, recovery, and death. Previous research on later-life health trajectories has emphasized that long observation windows are particularly important for detecting non-linear transitions, homeostasis, punctuated change, and mortality-related pathways [3,24].

A further data-quality issue warrants explicit mention. Fieldwork for the 2020 wave began face-to-face in late 2019 and was suspended in March 2020 following the introduction of nationwide COVID-19 lockdown measures. Data collection resumed between September and December of that year by telephone, yielding a smaller achieved sample and a change in survey mode for part of the wave [22]. This wave was included in the analysis, and the implications of this disruption are discussed among the limitations below.

The analytic sample included individuals aged 46–65 in 2004. This baseline age range was selected for two reasons. First, it allows the analysis to begin before most respondents reach older ages, making it possible to observe transitions from midlife into later life. Second, it provides sufficient variation in age at baseline to examine whether trajectory structures differ depending on the stage of the life course at which respondents enter the observation window. Respondents were divided into four five-year baseline age groups: 46–50, 51–55, 56–60, and 61–65 years. These age groups were analyzed separately because functional ability dynamics differ substantially depending on the age at which individuals enter the observation period. Table 1 presents descriptive statistics for the full analytic sample, including distributions by sex, education, and functional ability status at baseline and ending year, across these age groups.

**Table 1:** Study samples and descriptive statistics at baseline (2004).

	Age group 1 N = 743 (27.7%)	Age group 2 N = 711 (26.5%)	Age group 3 N = 657 (24.5%)	Age group 4 N = 570 (21.3%)
**Sex**				
Women	54.6%	55.3%	49.8%	46.3%
Men	45.4%	44.7%	50.2%	53.7%
**Educational level**				
Primary education or none	44.3%	50.1%	55.1%	67.0%
Secondary education or more	55.7%	49.9%	44.9%	33.0%
**Functional limitation status in baseline year (2004)**				
No limitations	95.2%	95.9%	91.9%	89.0%
Low limitations	2.6%	2.7%	4.0%	5.6%
Middle limitations	0.5%	0.1%	0.3%	0.7%
High limitations	1.7%	1.3%	3.8%	4.7%
**Functional limitation status in ending year (2020)**				
No limitations	70.4%	63.0%	41.4%	23.0%
Low limitations	8.1%	7.9%	11.0%	8.8%
Middle limitations	0.7%	0.7%	0.3%	1.2%
High limitations	8.9%	10.1%	11.9%	8.3%
Deceased	12.0%	18.3%	35.5%	58.8%

Note: Age group 1 = Individuals aged 46–50 in 2004. Age group 2 = Individuals aged 51–55 in 2004. Age group 3 = individuals aged 56–60 in 2004. Age group 4 = individuals aged 61–65 in 2004.

From the initial pool of eligible respondents, we retained individuals with no more than one missing functional ability observation across the six waves. This criterion was used to preserve the integrity of sequence reconstruction while retaining a broad analytic sample. For respondents with one missing functional ability observation, missing states were imputed using multiple imputation by chained equations with predictive mean matching, incorporating age, sex, educational attainment, and adjacent functional ability states as predictors. To assess the robustness of our findings to the imputation strategy, we re-estimated the sequence and cluster analysis on the subsample of individuals with fully complete functional ability information across all six waves, excluding all imputed observations (S1 Appendix). As reported there, the imputation of a single missing wave did not materially affect the trajectory typology where its effect could be isolated from sample-size reduction.

The final sample included 2,681 individuals and 16,086 person-wave observations. Individuals who died during the observation period were retained in the analysis, and death was treated as a functional ability state. This decision follows previous trajectory-based research that incorporates mortality into the study of later-life functional dynamics [3,4].

The analytic sample is defined by observability of functional ability status across the six waves and is therefore representative of respondents who remained in the panel rather than of the Chilean population aged 46–65 in 2004. S2 Table compares the analytic sample with the broader sample that includes individuals with missing observations and with a sample excluding deceased individuals. Differences between the analytic sample and the broader sample are modest and consistent in direction. Specifically, the analytic sample contains between 3.4 and 5.0 percentage points more respondents with primary education or less across the four age groups, a somewhat higher proportion of women in the three younger age groups, and a marginally lower baseline prevalence of no limitations in three of the four age groups. Attrition therefore does not appear to have retained a more advantaged or healthier subset of respondents. Retaining individuals who died during the observation period, and treating death as a functional ability state, further limits one of the principal sources of selective loss in long panels of aging populations.

### Functional ability measure

Functional ability was measured using self-reported difficulties in activities of daily living available in the EPS. We constructed a five-state categorical measure based on limitations in walking long distances, bathing, dressing, eating, and getting out of bed. The states were classified as follows: no limitation, low limitation, middle limitation, high limitation, and deceased.

The hierarchical classification reflects increasing severity in functional limitation. Respondents reporting no difficulty in the activities considered were classified as having no limitation. Low limitation captured difficulty walking long distances. Middle limitation captured difficulty bathing. High limitation captured difficulty with dressing, eating, or getting out of bed. As noted above, the deceased state was included to avoid treating mortality as missing information and to represent trajectories that end in death as part of the observed functional process.

This classification follows previous work on functional ability trajectories in Chile, where limitations in activities of daily living were used to reconstruct long-term functional ability states [4]. It is also consistent with broader research on functional ability and intrinsic capacity, which has emphasized the relevance of hierarchical and multidimensional approaches to later-life functioning [25]. As a sensitivity analysis, we re-estimated trajectories using the number of reported limitations instead of the hierarchical state variable (S3 Appendix). As reported there, the broad trajectory structure and the age-graded shift from sustained stability toward death-related trajectories were broadly preserved, although concordance decreased with baseline age.

### Sex and educational attainment

Sex was measured using the variable available in the EPS and categorized as women and men. Following the Sex and Gender Equity in Research guidelines, we refer to sex when describing this variable because the survey records sex rather than gender identity [26].

Educational attainment was categorized as primary education or less versus secondary education or more. This classification reflects a relevant educational threshold for the cohorts under study and maintains sufficient sample size for trajectory comparisons within age groups. Education is commonly used in functional ability and disability research as an indicator of socioeconomic position and cumulative life-course resources [4,7–12]. In Chile, educational attainment differs substantially across cohorts, with older respondents more frequently concentrated in lower educational categories. This age-related educational composition was considered when interpreting differences across baseline age groups [20].

### Sequence analysis and trajectory classification

We used sequence analysis to reconstruct individual functional ability trajectories across six observation points from 2004 to 2020 [27]. Each respondent’s trajectory was represented as an ordered sequence of functional ability states. Sequence analysis is well suited for this study because it captures both the timing and ordering of transitions between functional states, including stability, decline, recovery, fluctuation, and death. This method has been increasingly used in life-course research because it allows researchers to analyze trajectories as whole sequences rather than as isolated transitions or single outcomes [28].

For each baseline age group, we calculated pairwise dissimilarities between individual sequences using optimal matching. Substitution costs were set to 2, and hierarchical clustering using Ward’s method was applied to the resulting dissimilarity matrix. Cluster solutions were evaluated using multiple criteria, including average silhouette width, point biserial correlation, Hubert’s Gamma, and Hubert’s C. Fig 1 presents the normalized scores (Z-scores) for each criterion for each age group. Based on these metrics and guided by theoretical considerations [29], we selected the number of clusters that best captured the heterogeneity of functional ability trajectories within each age group. The gray dashed vertical lines indicate the selected cluster solutions. Specifically, the optimal number of clusters was seven among individuals aged 46–50 at baseline, six among those aged 51–55, eight among those aged 56–60, and six among those aged 61–65.

**Fig 1:**
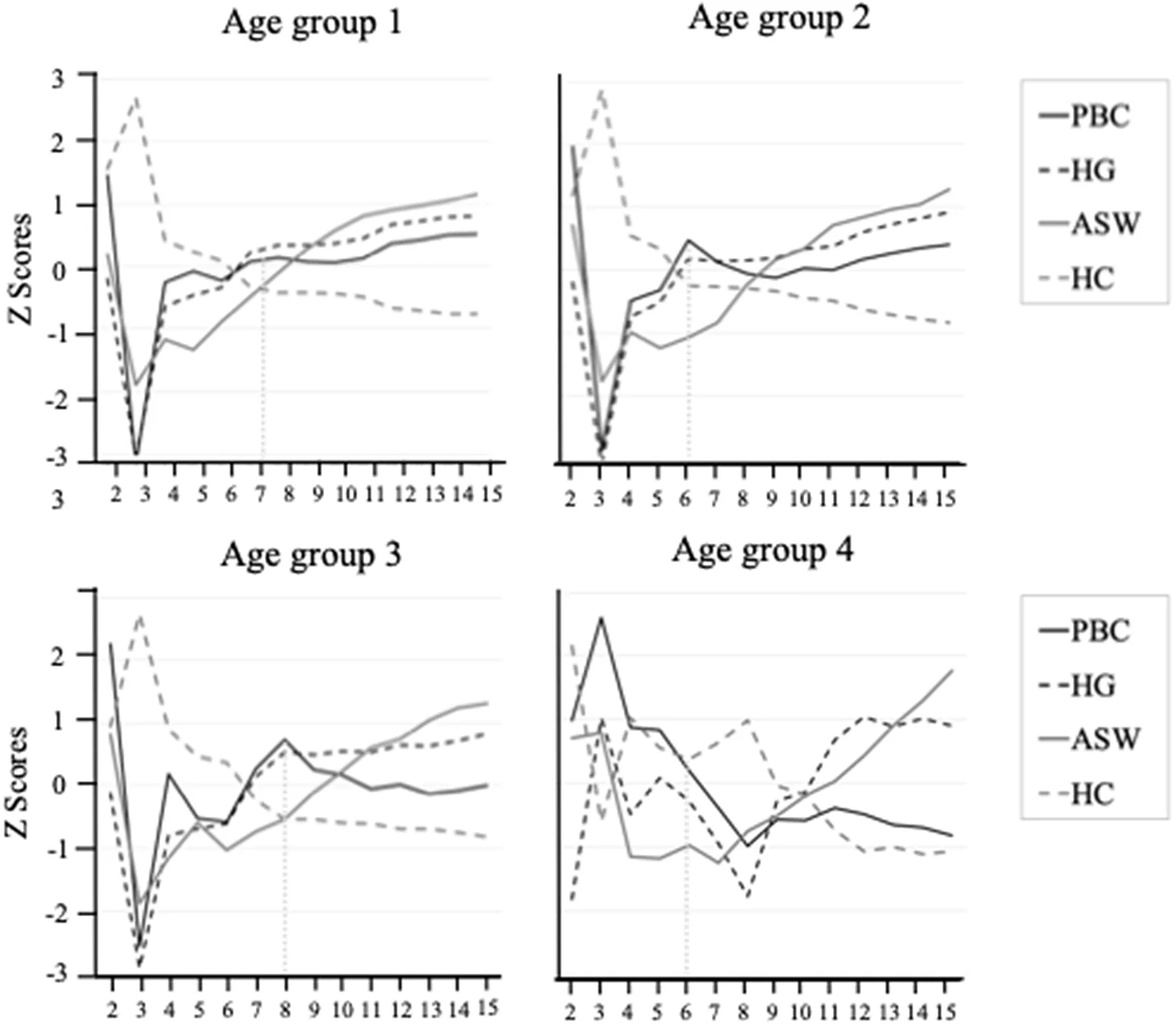
Cluster cut-off criteria across age groups. Note: Gray dashed vertical lines indicate the optimal number of representative trajectory clusters need to summarize the diversity of functional ability trajectories. PBC = point biserial correlation; HG = Hubert’s Gamma; ASW = average silhouette width; HC = Hubert’s C. Normalized scores shown. Hogher ASW, PCB, and HG values indicate better cluster solutions. Lower HC values indicate better cluster solutions.

### Analytical strategy

The analysis proceeded in four steps. First, we described baseline characteristics of the analytic sample by age group, including sex, educational attainment, and functional ability status. Second, we identified detailed functional ability trajectories within each age group using sequence analysis and cluster analysis. Third, we grouped the detailed trajectories into three broader trajectory types: mostly non-declining, progressive-declining, and equivocal-declining. Fourth, we examined whether membership in these trajectory types differed by sex and educational attainment, using chi-squared tests and referencing the composition of each trajectory type to the overall sex and educational composition of its baseline age group, so that departures from the marginal distribution can be distinguished from compositions that reproduce it. All analyses were conducted in R [30], using the TraMineR [29] and mice [31] packages.

Analyses were conducted without survey weights. This decision follows from the design of the study in three respects. The construction of the typology rests on pairwise dissimilarities between sequences and on hierarchical clustering, procedures into which expansion factors cannot be incorporated in a straightforward manner at the stage where trajectory types are defined. The substantive claims concern the sex and educational composition of shared trajectory categories within each baseline age group, a within-sample comparison for which weighting is less consequential than it would be for national prevalence estimation. Previous research using the same survey and a comparable trajectory design reported highly similar results with and without the expansion factor, which is consistent with an accurate sampling strategy and adequate fieldwork in the EPS [4].

### Ethics statement

This study used the EPS, a publicly available secondary dataset. The study respected the original consent and confidentiality procedures under which the EPS data were collected and made available.

## Results

### Overview of the trajectory typologies

The sequence analysis identified a set of functional ability trajectories within each baseline age group. These trajectories were estimated using the full age-group-specific samples and were then grouped into three broader trajectory types: mostly non-declining, progressive-declining, and equivocal-declining. This analytical strategy allows comparisons by sex and education to be made after trajectory types were defined within the pooled sample of each age group. Importantly, the grouping into three broad types corresponds to an interpretive harmonization based on the qualitative shape of the detailed trajectories, namely the presence, ordering, and timing of transitions toward more severe limitation states or death. This grouping is intended to support cross-age description and does not imply that the underlying detailed clusters are statistically equivalent across age groups.

The mostly non-declining type includes trajectories characterized by sustained absence of functional limitations or preserved functional ability before death. The progressive-declining type includes trajectories marked by a more ordered movement from no or low limitation toward more severe limitation or death. The equivocal-declining type includes trajectories characterized by non-linear, unstable, or fluctuating functional ability patterns, including movements across limitation states and, in some cases, partial recovery. Fig 2 presents the detailed trajectories composing each of the three broader types for each age group using sequence index plots. In these plots, the X-axis represents age and each horizontal line represents one individual trajectory. The plots therefore show intra-individual transitions between functional ability states over time.

**Fig 2:**
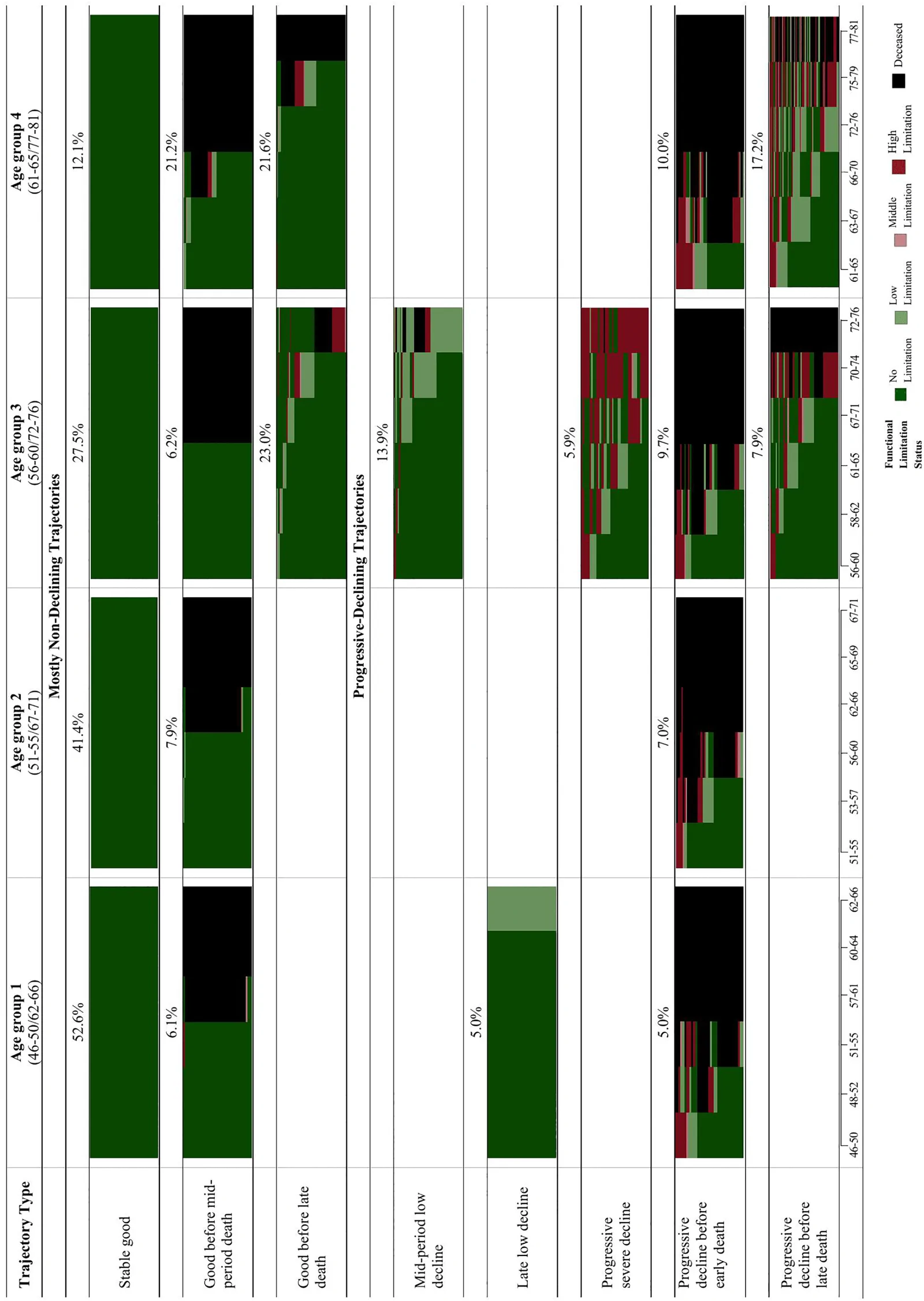
Functional ability trajectory types across four age groups.

### Functional ability trajectories across age groups

As shown in Fig 2, mostly non-declining trajectories represented a large share of respondents across the four age groups, but their internal composition changed with baseline age. Among individuals aged 46–50 at baseline, 58.7% were classified in mostly non-declining trajectories. This type was composed mainly of the *stable good* trajectory, which represented 52.6% of the cohort, and the *good before mid-period death* trajectory, which represented 6.1%. Progressive-declining trajectories represented 10.0% of the cohort and were composed of *late low decline* and *progressive decline before early death*, each accounting for 5.0%. Equivocal-declining trajectories represented 31.4%, including *early severe decline* at 7.3%, *late severe decline* at 10.4%, and *equivocal recovery* at 13.7%.

Among individuals aged 51–55 at baseline, mostly non-declining trajectories represented 49.3% of the cohort. This type included *stable good* at 41.4% and *good before mid-period death* at 7.9%. Progressive-declining trajectories represented 7.0%, corresponding to the single trajectory *progressive decline before early death*. Equivocal-declining trajectories represented 43.8%, composed of *early severe decline* at 5.9%, *late severe decline* at 11.7%, and *equivocal recovery* at 26.2%. This age group therefore shows the highest prevalence of equivocal-declining trajectories across the four groups.

Among individuals aged 56–60 at baseline, mostly non-declining trajectories represented 56.7% of the cohort. This type included *stable good* at 27.5%, *good before mid-period death* at 6.2%, and *good before late death* at 23.0%. Progressive-declining trajectories represented 37.4%, making this the age group with the largest progressive-declining component. This broad type included *mid-period low decline* at 13.9%, *progressive severe decline* at 5.9%, *progressive decline before early death* at 9.7%, and *progressive decline before late death* at 7.9%. Equivocal-declining trajectories represented 5.8%, corresponding to the single trajectory *early low decline*.

Among individuals aged 61–65 at baseline, mostly non-declining trajectories represented 54.9% of the cohort. This type included *stable good* at 12.1%, *good before mid-period death* at 21.2%, and *good before late death* at 21.6%. Progressive-declining trajectories represented 27.2%, composed of *progressive decline before early death* at 10.0% and *progressive decline before late death* at 17.2%. Equivocal-declining trajectories represented 17.9%, corresponding to the trajectory *late severe decline*.

### Sex differences in trajectory types across age groups

As shown in Table 2, sex differences in trajectory membership were statistically significant in all four age groups when considering both the three main trajectory types and the detailed trajectories. Specifically, for the three main types, the association between sex and trajectory membership was statistically significant in age group 1, χ^2^ = 17.27, p < .001; age group 2, χ^2^ = 15.14, p < .001; age group 3, χ^2^ = 8.08, p = .018; and age group 4, χ^2^ = 24.97, p < .001.

**Table 2:** Sex composition of main and detailed functional ability trajectory types by age group.

	Age group 1 (46-50/62-66)			Age group 2 (51-55/67-71)			Age group 3 (56-60/72-76)			Age group 4 (61-65/77-81)		
	Men (%)	Women (%)	–	Men (%)	Women (%)	–	Men (%)	Women (%)	–	Men (%)	Women (%)	–
**Age Group Composition** **(All Respondents)**	45.4	54.6	–	44.7	55.3	–	50.2	49.8	–	53.7	46.3	–
**Main Trajectory Types**	**Men** **(%)**	**Women** **(%)**	**P value**	**Men** **(%)**	**Women** **(%)**	**P value**	**Men** **(%)**	**Women** **(%)**	**P value**	**Men** **(%)**	**Women** **(%)**	**P value**
Mostly Non-Declining Trajectories	51.6	48.4	< .001	50.3	49.7	< .001	54.7	45.3	.018	62.3	37.7	< .001
Progressive-Declining Trajectories	40.5	59.5		56.0	44.0		43.1	56.9		48.4	51.6	
Equivocal-Declining Trajectories	35.2	64.8		36.7	63.3		52.6	47.4		35.3	64.7	
**Detailed Trajectory Types**	**Men** **(%)**	**Women** **(%)**	**P value**	**Men** **(%)**	**Women** **(%)**	**P value**	**Men** **(%)**	**Women** **(%)**	**P value**	**Men** **(%)**	**Women** **(%)**	**P value**
Stable good	49.9	50.1	< .001	47.6	52.4	< .001	61.3	38.7	< .001	46.4	53.6	< .001
Good before mid-period death	66.7	33.3		64.3	35.7		63.4	36.6		57.9	42.1	
Good before late death	–	–		–	–		44.4	55.6		75.6	24.4	
Mid-period low decline	–	–		–	–		38.5	61.5		–	–	
Late low decline	29.7	70.3		–	–		–	–		–	–	
Progressive severe decline	–	–		–	–		20.5	79.5		–	–	
Progressive decline before early death	51.4	48.6		56.0	44.0		56.2	43.8		66.7	33.3	
Progressive decline before late death	–	–		–	–		51.9	48.1		37.8	62.2	
Early low decline	–	–		–	–		52.6	47.4		–	–	
Early severe decline	22.2	77.8		28.6	71.4		–	–		–	–	
Late severe decline	33.8	66.2		38.6	61.4		–	–		35.3	64.7	
Equivocal recovery	43.1	56.9		37.6	62.4		–	–		–	–	

Note: Percentages indicate the composition of each trajectory type by sex. The first row reports the sex composition of all respondents in each baseline age group, so that trajectory-specific compositions can be read as departures from the marginal distribution. Chi-squared tests evaluate whether trajectory membership differs by sex within each age group. P-values of chi-squared tests are reported.

Interpreting these compositions requires reference to the overall sex composition of each baseline age group. Women constitute 54.6% of respondents aged 46–50 at baseline and 55.3% of those aged 51–55, whereas men constitute 50.2% of those aged 56–60 and 53.7% of those aged 61–65 (Table 1). Table 2 reports these marginal distributions alongside the trajectory-specific compositions, and the description below expresses each composition as a departure in percentage points from the marginal proportion of the corresponding age group. A departure is described as reversing the aggregate composition when the majority within a trajectory type differs from the majority in the age group as a whole, and as reinforcing it when the two coincide.

Mostly non-declining trajectories had a higher proportion of men than women in all four age groups, although the margin was minimal in age group 2. In age group 1, mostly non-declining trajectories were composed of 51.6% men and 48.4% women, which reverses the aggregate composition of an age group in which women predominate, departing by 6.2 percentage points from the marginal proportion of men. The same holds in age group 2, where a composition of 50.3% men and 49.7% women, although close to parity in absolute terms, departs by 5.6 percentage points from a marginal of 44.7% men and therefore also reverses the aggregate composition. In age groups 3 and 4, the male majority in this type, 54.7% and 62.3% respectively, reinforces an aggregate composition in which men already predominate, with departures of 4.5 and 8.6 percentage points from the corresponding marginals.

Equivocal-declining trajectories were composed of a higher proportion of women in age groups 1, 2, and 4. In age group 1, this type was composed of 35.2% men and 64.8% women, and in age group 2 of 36.7% men and 63.3% women; both compositions reinforce a female-majority aggregate distribution, with departures of 10.2 and 8.0 percentage points. In age group 4, a composition of 35.3% men and 64.7% women reverses an aggregate composition in which men constitute 53.7% of respondents, and represents the largest single departure in Table 2, at 18.4 percentage points. In age group 3, the composition of this type, 52.6% men and 47.4% women, closely reproduces a nearly balanced marginal, departing by 2.4 percentage points.

Progressive-declining trajectories showed more variation across age groups. Women represented the larger share in age groups 1, 3, and 4, while men represented the larger share in age group 2. In age group 1, the female share of 59.5% reinforces a female-majority aggregate composition, exceeding the marginal by 4.9 percentage points. The remaining three departures reverse the aggregate composition of their age group: 56.0% men in age group 2, exceeding a marginal of 44.7% by 11.3 percentage points; 56.9% women in age group 3, exceeding a marginal of 49.8% by 7.1 percentage points; and 51.6% women in age group 4, exceeding a marginal of 46.3% by 5.3 percentage points. Overall, the concentration of women in equivocal-declining trajectories is the most consistent pattern across age groups, and it is most pronounced in age group 4, where it inverts the aggregate sex composition of the age group.

### Educational differences in trajectory types across age groups

Table 3 indicates educational differences in trajectory membership. The association between educational level and the three main trajectory types was statistically significant in the first three age groups: age group 1, χ^2^ = 11.00, p = .004; age group 2, χ^2^ = 9.29, p = .010; and age group 3, χ^2^ = 8.06, p = .018. In age group 4, the association was not statistically significant, although the p-value approached conventional thresholds, χ^2^ = 5.07, p = .079. For the detailed trajectories, education was significantly associated with trajectory membership in age groups 1, 2, and 3, but not in age group 4.

**Table 3:** Educational composition of main and detailed functional ability trajectory types by age group.

	Age group 1 (46-50/62-66)			Age group 2 (51-55/67-71)			Age group 3 (56-60/72-76)			Age group 4 (61-65/77-81)		
	Low ed (%)	High ed (%)	–	Low ed (%)	High ed (%)	–	Low ed (%)	High ed (%)	–	Low ed (%)	High ed (%)	–
**Age Group Composition** **(All Respondents)**	44.3	55.7	–	50.1	49.9	–	55.1	44.9	–	67.0	33.0	–
**Main Trajectory Types**	**Low ed** **(%)**	**High ed** **(%)**	**P value**	**Low ed** **(%)**	**High ed** **(%)**	**P value**	**Low ed** **(%)**	**High ed** **(%)**	**P value**	**Low ed** **(%)**	**High ed** **(%)**	**P value**
Mostly Non-Declining Trajectories	40.1	59.9	.004	44.3	55.7	.010	50.4	49.6	.018	63.9	36.1	.079
Progressive-Declining Trajectories	40.5	59.5		54.0	46.0		60.6	39.4		74.2	25.8	
Equivocal-Declining Trajectories	53.2	46.8		55.9	44.1		65.8	34.2		65.7	34.3	
**Detailed Trajectory Types**	**Low ed** **(%)**	**High ed** **(%)**	**P value**	**Low ed** **(%)**	**High ed** **(%)**	**P value**	**Low ed** **(%)**	**High ed** **(%)**	**P value**	**Low ed** **(%)**	**High ed** **(%)**	**P value**
Stable good	37.9	62.1	< .001	41.8	58.2	.008	40.3	59.7	< .001	59.4	40.6	.246
Good before mid-period death	60.0	40.0		57.1	42.9		65.9	34.1		67.8	32.2	
Good before late death	–	–		–	–		58.3	41.7		62.6	37.4	
Mid-period low decline	–	–		–	–		58.2	41.8		–	–	
Late low decline	21.6	78.4		–	–		–	–		–	–	
Progressive severe decline	–	–		–	–		61.5	38.5		–	–	
Progressive decline before early death	59.5	40.5		54.0	46.0		57.8	42.2		75.4	24.6	
Progressive decline before late death	–	–		–	–		67.3	32.7		73.5	26.5	
Early low decline	–	–		–	–		65.8	34.2		–	–	
Early severe decline	59.3	40.7		54.8	45.2		–	–		–	–	
Late severe decline	46.8	53.2		62.7	37.3		–	–		65.7	34.3	
Equivocal recovery	54.9	45.1		53.2	46.8		–	–		–	–	

Note: “Low ed” = primary education or less. “High ed” = secondary education or more. Percentages indicate the composition of each trajectory type by education. The first row reports the educational composition of all respondents in each baseline age group, so that trajectory-specific compositions can be read as departures from the marginal distribution. Chi-squared tests evaluate whether trajectory membership differs by educational level within each age group. P-values of chi-squared tests are reported.

As with sex, the educational composition of the trajectory types requires reference to the marginal distribution of each age group. Respondents with primary education or less constitute 44.3% of age group 1, 50.1% of age group 2, 55.1% of age group 3, and 67.0% of age group 4 (Table 1). Table 3 reports these marginals alongside the trajectory-specific compositions.

Mostly non-declining trajectories had a higher proportion of individuals with secondary education or more in age groups 1 and 2. In age group 1, this type was composed of 59.9% individuals with secondary education or more and 40.1% individuals with primary education or less, a departure of 4.2 percentage points below the marginal proportion with primary education or less. In age group 2, the corresponding figures were 55.7% and 44.3%, a departure of 5.8 percentage points below the marginal. In age group 3, the mostly non-declining type was nearly balanced by education, with 49.6% individuals with secondary education or more and 50.4% individuals with primary education or less, which departs by 4.7 percentage points below a marginal of 55.1% and therefore reverses a low-education aggregate majority into near parity. In age group 4, mostly non-declining trajectories were composed mainly of individuals with primary education or less, 63.9%, a figure that falls 3.1 percentage points below the marginal of 67.0% and therefore reproduces the educational composition of this older age group.

Progressive-declining trajectories became increasingly composed of lower-educated respondents across age groups. The proportion of individuals with primary education or less within progressive-declining trajectories increased from 40.5% in age group 1 to 54.0% in age group 2, 60.6% in age group 3, and 74.2% in age group 4. Referenced to the marginals, these figures correspond to a departure of 3.8 percentage points below the age-group composition in age group 1 and to departures of 3.9, 5.5, and 7.2 percentage points above it in age groups 2, 3, and 4. The educational gradient in this trajectory type therefore strengthens with baseline age both in absolute terms and relative to the composition of each age group.

Equivocal-declining trajectories were composed of 53.2% respondents with primary education or less in age group 1, 55.9% in age group 2, 65.8% in age group 3, and 65.7% in age group 4. Referenced to the marginals, the departures are 8.9, 5.8, and 10.7 percentage points above the age-group composition in the first three groups, and the composition in age group 1 reverses an aggregate distribution in which respondents with secondary education or more constitute 55.7% of the age group. In age group 4, by contrast, the composition falls 1.3 percentage points below the marginal, so that the apparent concentration of lower-educated respondents in this trajectory type in the oldest age group reflects the educational composition of the age group itself. More generally, all three trajectory types in age group 4 fall within 7.2 percentage points of the marginal educational distribution, which is consistent with the absence of a statistically significant association in that age group. Taken together, educational differences are most visible in the composition of equivocal-declining trajectories in the three younger age groups and in progressive-declining trajectories in the two older age groups.

## Discussion

This study examined long-term functional ability trajectories among Chilean adults aged 46–65 at baseline over a 17-year period. Its contribution is both substantive and methodological. Substantively, the findings show that functional aging in Chile unfolds through heterogeneous pathways that can be summarized as mostly non-declining, progressive-declining, and equivocal-declining trajectories. Methodologically, the analysis derives trajectory typologies within pooled age-group samples before comparing sex and educational groups, and references the composition of each trajectory type to the marginal composition of its age group.

The findings reinforce the value of treating functional ability as a dynamic process, since capturing functional aging requires more than a single contrast between limitation and no limitation at one point in time. The three broad trajectory types identified in this study reflect different temporal organizations of functional ability: sustained or preserved functioning, ordered functional deterioration, and unstable or equivocal transitions between functional states. This is consistent with the broader shift in disability and aging research from static descriptions toward longitudinal analyses of functional change.

One important implication of the results is that the same broad trajectory type can have different meanings depending on baseline age, emphasizing the benefits of early prevention [32]. Mostly non-declining trajectories were prevalent across the four age groups, but their internal composition changed substantially. In younger age groups, mostly non-declining trajectories mainly captured sustained functional stability. In older age groups, this category increasingly captured preserved functional ability before death. Both variants can be read as forms of healthy aging, understood as preserving function for as long as possible, but they are not interchangeable. In younger cohorts, it often describes uninterrupted functional stability over the observation window. In older cohorts, it may describe a pathway in which individuals remain functionally able for several waves before mortality becomes part of the trajectory. Longitudinal data are therefore essential for distinguishing sustained functional stability from preserved functioning before death.

The progressive-declining trajectories were especially visible from the late-fifties baseline group onward. This suggests that individuals aged 56–60 at baseline occupy a relevant position for observing ordered functional deterioration over a 17-year period. At this stage of the life course, respondents are old enough for functional limitations and mortality to become visible during follow-up, while still young enough for the ordering and timing of decline to be observed before the end of the panel. This finding contributes to the literature by showing that the visibility of progressive decline depends on where individuals are in the life course when longitudinal observation begins. The result also supports the value of sequence analysis for aging research because it captures the timing and order of functional transitions rather than reducing functional ability to a baseline status or an end-point outcome. Additionally, results support the importance of rehabilitation and early prevention as relevant strategies to improve functional ability in the population [33,34].

Sex differences in trajectory membership were evident across all age groups, but the form of these differences varied by baseline age. Men were generally more represented in mostly non-declining trajectories, especially in age groups where preserved functioning before death formed part of this category. Women were more represented in declining trajectories, particularly equivocal-declining trajectories in age groups 1, 2, and 4, and progressive-declining trajectories in age group 3. This suggests that sex differences in functional aging are expressed through the form and temporal organization of decline. Women’s higher representation in equivocal- and progressive-declining trajectories indicates greater concentration in pathways involving instability, severe limitation, or ordered deterioration, whereas men’s higher representation in mostly non-declining trajectories partly reflects pathways of sustained functioning and, in older cohorts, preserved functioning before death.

Referencing these compositions to the marginal distribution of each age group clarifies which of them constitute genuine departures from the aggregate composition and which reproduce it. Two configurations stand out. In the two younger age groups, where women form the majority of respondents, the male majority in mostly non-declining trajectories reverses the aggregate composition, which makes the association between sex and sustained functioning most clearly visible precisely where the aggregate distribution works against it. In the oldest age group, where men form the majority, the concentration of women in equivocal-declining trajectories reverses the aggregate composition by 18.4 percentage points, the largest departure observed. Conversely, the male majority in mostly non-declining trajectories in the two older age groups reinforces an already male-majority distribution, so that its interpretation depends on the magnitude of the departure rather than on the direction of the difference alone. The educational findings follow a comparable logic and require the same caution. Specifically, the concentration of respondents with primary education or less in equivocal-declining trajectories is a substantive departure in the three younger age groups, whereas in the oldest age group all three trajectory types track the marginal educational distribution closely, which accounts for the absence of a statistically significant association without implying an absence of educational disadvantage in that cohort.

Educational differences also contribute to the social patterning of functional ability trajectories. In the first three age groups, education was significantly associated with grouped trajectory membership, while in the oldest age group the association was neither statistically significant nor discernible once composition is referenced to the age-group marginal. This pattern suggests that education differentiates functional trajectories from midlife through the transition into older age, while in the oldest cohort the wider spread of mortality and functional limitation across educational groups, combined with a marginal distribution in which two thirds of respondents have primary education or less, makes the association less statistically distinguishable. The educational gradient was especially evident in declining trajectories. Respondents with primary education or less were more concentrated in progressive- and equivocal-declining trajectories across most age groups, while respondents with secondary education or more were more represented in mostly non-declining trajectories in the two youngest age groups.

These findings are consistent with international evidence showing that education is associated with disability, physical functioning, and later-life health trajectories [7–13]. Education can be interpreted as a life-course marker of accumulated social, material, and cognitive resources. In the Chilean context, where older cohorts experienced unequal access to schooling and where health and social protection resources remain stratified, educational attainment is a relevant marker for understanding inequality in functional aging.

These results can also be read against the contextual expectations set out at the outset. The concentration of women and of respondents with primary education or less in equivocal-declining trajectories is consistent with a setting in which recovery from an episode of limitation depends substantially on household resources, given a segmented health system and limited formal long-term care provision [21]. The strengthening of the educational gradient in progressive-declining trajectories with baseline age is consistent with cumulative exposure to physically demanding and unprotected work among lower-educated individuals in a labor market characterized by weak employment protection and persistent informality [19].

At the same time, the educational composition of the analytic sample shifts markedly across baseline age groups, rising from 44.3% with primary education or less in the youngest group to 67.0% in the oldest, so that educational differences observed here operate alongside a rapidly changing cohort composition and cannot be read as a pure period or age effect [20]. The heterogeneity of the trajectories themselves, encompassing sustained stability, ordered decline, and fluctuating patterns, resembles that reported in higher-income settings, which suggests that the distinctiveness of the Chilean case lies in how social groups are distributed across these pathways rather than in the existence of the pathways themselves.

The results also invite a careful interpretation of functional independence in later life. Cross-sectional estimates in Chile suggest that a large proportion of older adults live without functional dependence [21]. The longitudinal findings presented here add a different perspective. A person observed without functional limitations at one point in time may later remain stable, experience progressive decline, move through fluctuating states, or die after a period of preserved functioning. Cross-sectional estimates of functional independence therefore describe the prevalence of independence at a given moment while leaving sustained independence and transitional independence within a longer trajectory indistinguishable. This distinction is relevant for interpreting the demand for care and support because longitudinal trajectories reveal movements between functional states that cross-sectional measures cannot fully capture.

### Limitations

Some limitations should be acknowledged. First, although the 17-year observation window is a major strength, the intervals between survey waves may miss short-term transitions in functional ability. More frequent observations would allow for a more detailed understanding of the timing, duration, and reversibility of functional changes [24]. This is particularly relevant for equivocal-declining trajectories, where shorter-term movements between limitation states may be partially unobserved.

Second, the inclusion criteria required respondents to have no more than one missing functional ability observation. This strategy supports robust sequence reconstruction, but it may exclude individuals with more unstable survey participation. The analytic sample is therefore defined by observability across the six waves and reflects the cumulative retention of a long panel. Its composition is broadly comparable to that of the wider eligible sample and, if anything, slightly less advantaged in educational and functional terms, which is reassuring for a study concerned with functional decline. Nonetheless, individuals lost to follow-up for reasons correlated with unobserved health deterioration cannot be recovered, and some selection bias cannot be ruled out.

Third, the final observation point requires a specific caveat. Fieldwork for the 2020 wave was interrupted by the COVID-19 pandemic and completed under a mixed-mode design, with part of the sample interviewed by telephone. Mode effects on the self-report of difficulties in activities of daily living cannot be ruled out, and the reduced achieved sample may have introduced additional selectivity at the endpoint of the observation window.

Three features of our design limit the leverage of this issue on our conclusions. First, trajectories are constructed from whole sequences of six observations, so that any single wave exerts bounded influence on cluster assignment. Second, the functional ability measure is a coarse hierarchical classification that is less sensitive to fine-grained reporting differences than a continuous score. And third, the sensitivity analysis based on the number of reported limitations reproduced the broad trajectory structure. Results concerning the final period should nonetheless be interpreted with this caveat in mind.

Fourth, analyses were conducted without survey weights, for the reasons stated in the analytical strategy. The prevalence figures reported for each trajectory type therefore describe the analytic sample and should not be read as population estimates for Chilean adults aged 46–65 in 2004. The compositional comparisons that support our substantive claims are internal to each age group and are less affected by this decision than prevalence estimation would be.

Fifth, functional ability was classified using a hierarchical index based on self-reported limitations in selected activities of daily living. This approach follows prior research on functional ability trajectories in Chile [4], but it does not incorporate clinical scales such as the Barthel or Katz Index. This may limit direct comparability with studies using different measurement instruments [35]. At the same time, the use of repeated self-reported functional limitations allows the analysis to reconstruct long-term intra-individual trajectories in a nationally representative panel.

Sixth, a further consideration concerns comparability across baseline age groups. Within each age group, the pooled estimation ensures that sex and educational groups are compared within shared trajectory categories. Across age groups, however, the three broad trajectory types represent an interpretive harmonization of age-group-specific cluster solutions rather than a jointly estimated typology. Comparisons across baseline age groups should therefore be read as descriptive rather than as contrasts between strictly equivalent constructs.

Seventh, educational attainment was categorized as primary education or less versus secondary education or more. This binary classification is useful for maintaining sufficient sample size within age groups, but it may obscure differences within educational strata, especially among respondents with tertiary education. Future research could examine whether more detailed educational categories modify the distribution of individuals across mostly non-declining, progressive-declining, and equivocal-declining trajectories.

Finally, the study focused on sex and education as two central dimensions of social inequality. Other dimensions, including income, occupation, ethnicity, region, caregiving histories, household composition, and access to health services, may also shape functional ability trajectories. Future research could examine how these factors combine across the life course and whether they help explain why some groups are more concentrated in progressive or equivocal forms of decline.

## Conclusion

This study examined long-term functional ability trajectories among Chilean adults observed from midlife into older age. By estimating trajectory typologies within pooled age-group samples, the analysis provides a methodologically comparable account of how functional ability unfolds across socially differentiated populations. The findings show that functional aging is organized through multiple temporal patterns of continuity, deterioration, instability, and mortality-related transition. Mostly non-declining, progressive-declining, and equivocal-declining trajectories capture these different forms across the life course.

By focusing on Chile, this study extends functional ability trajectory research to a Latin American context characterized by rapid population aging and marked social inequality. The results contribute to previous Chilean evidence on functional ability trajectories and show that sex and education remain relevant dimensions for understanding how later-life functioning develops over time. They also suggest that aging research benefits from examining the composition and internal meaning of trajectory types, and from referencing that composition to the aggregate composition of the population under study, rather than treating broad categories such as stability or decline as equivalent across age groups.

The ways aging is described shape how aging is understood [36]. The trajectories documented here offer a longitudinal description of aging as heterogeneous and socially patterned. Functional ability changes through diverse pathways, and these pathways are connected to social position across the life course. Recognizing this heterogeneity allows research on aging to move beyond static contrasts between dependence and independence and toward a more dynamic understanding of functional ability, inequality, and later-life health.

Declaration of Generative AI and AI-assisted technologies in the writing process: We acknowledge the use of artificial intelligence to improve grammar, clarity, and the flow of writing.

## Supporting information

S1 Appendix Sensitivity Analysis 1. (DOCX)

S2 Table Comparison of key variables in the study sample, a sample with missing observations, and a sample excluding deceased individuals. (DOCX)

S3 Appendix Sensitivity Analysis 2. (DOCX)
