# Supplementary material for: Sex and educational inequalities across functional ability trajectories in Chile: A sequence analysis of a 17-year national panel
Source: PLoS One. 2026 Nov 3;21(11):e0361826. doi: 10.1371/journal.pone.0361826 (PMC13730331; doi:10.1371/journal.pone.0361826)
Supplement: Supplementary file 1 [file pone.0361826.s001.docx]

**S1 Appendix**. Sensitivity Analysis 1

To assess the robustness of our findings to the imputation strategy, we re-estimated the sequence and cluster analysis on the subsample of individuals with fully complete functional ability information across all six waves (excluding all imputed observations), and cross-classified each individual’s original and complete-case broad trajectory-type assignments (see Supplementary Figure 1 and Supplementary Table 2). In the two younger age groups, where complete-case restriction reduced the sample only modestly, the two solutions assigned 100% of individuals to the same broad trajectory type (Cohen’s κ = 1.00, Adjusted Rand index for the broad types = 1.00), indicating that the imputation of a single missing wave did not alter the typology. In the two older age groups, where higher mortality meant that the complete-case subsample was substantially smaller, concordance was lower (agreement of 60.6% and 75.9%, Cohen’s κ = 0.31 and 0.63). This reduction reflects the combined effect of the smaller sample and the re-estimation of the cluster structure on that smaller sample, rather than the imputation procedure alone, and was most pronounced in the 56–60 group, where trajectories involving preserved functioning before death and decline-related trajectories are densely concentrated and the boundary between them is more sensitive to sample composition. The broad three trajectory types remained identifiable in all age groups (Supplementary Figure 1). Overall, the imputation of a single missing wave did not materially affect the trajectory typology where its effect could be isolated from sample-size reduction.

| **Trajectory Type** | **Age group 1**  (46-50/62-66) | **Age group 2**  (51-55/67-71) | **Age group 3**  (56-60/72-76) | **Age group 4**  (61-65/77-81) |
| --- | --- | --- | --- | --- |
|  | **Mostly Non-Declining Trajectories** | | | |
|  | 38.6% | 28.2% | 25.9% | 12.0% |
| Stable good |  |  |  |  |
|  |  |  | 7.1% |  |
| Good before  early death |  |  |  |  |
|  | 13.7% | 13.6% | 12.9% | 30.2% |
| Good before  mid-period death |  |  |  |  |
|  | **Progressive-Declining Trajectories** | | | |
|  | 4.9% |  | 18.5% |  |
| Late low decline |  |  |  |  |
|  |  |  | 10.6% |  |
| Progressive severe decline |  |  |  |  |
|  | 11.2% | 13.6% | 4.1% | 23.8% |
| Progressive decline before early death |  |  |  |  |
|  |  |  | 8.5% |  |
| Progressive decline before late death |  |  |  |  |
|  |  |  |  |  |

**Figure S1.1**. Functional ability trajectory types across four age groups excluding individuals with missing observations

**Figure S1.1**. Functional ability trajectory types across four age groups excluding individuals with missing observations (continued)

|  | **Age group 1**  (46-50/62-66) | | **Age group 2**  (51-55/67-71) | | **Age group 3**  (56-60/72-76) | | **Age group 4**  (61-65/77-81) |
| --- | --- | --- | --- | --- | --- | --- | --- |
| **Trajectory Type** | **Equivocal-Declining Trajectories** | | | | | | |
|  | 6.7% | 6.5% | |  | | 4.9% | |
| Early severe decline |  |  | |  | |  | |
|  | 12.6% | 16.9% | | 12.4% | | 29.0% | |
| Late severe decline |  |  | |  | |  | |
|  | 12.3% | 21.1% | |  | |  | |
| Equivocal recovery |  |  | |  | |  | |
|  |  |  | |  | |  | |

| **Baseline age group** | **N** | **Agreement on broad trajectory types (%)** | **Cohen’s κ (broad trajectory types)** | **Adjusted Rand Index (broad trajectory types)** | **Adjusted Rand Index (detailed trajectory types)** |
| --- | --- | --- | --- | --- | --- |
| Age group 1 | 284 | 100.0% | 1.00 | 1.00 | 0.892 |
| Age group 2 | 337 | 100.0% | 1.00 | 1.00 | 0.936 |
| Age group 3 | 340 | 60.6% | 0.31 | 0.15 | 0.678 |
| Age group 4 | 324 | 75.9% | 0.63 | 0.47 | 0.593 |

**Table S1.1.** Concordance between the main trajectory typology (imputing one missing observation) and the sensitivity-analysis typology (excluding individuals with missing observations), by baseline age group

Notes: This table reports the concordance between the main trajectory typology, estimated on the full analytic sample (which retained individuals with up to one imputed functional ability observation), and a typology re-estimated on the subsample of individuals with fully complete functional ability information across all six waves (excluding all imputed observations). Because the complete-case individuals are a subset of the main analytic sample, each carries both an original and a complete-case trajectory assignment, allowing the two solutions to be cross-classified at the individual level.

N = number of matched complete cases. Agreement = percentage of individuals assigned to the same broad trajectory type (mostly non-declining, progressive-declining, equivocal-declining) under both solutions. Cohen’s κ = chance-corrected agreement on the three broad types. Adjusted Rand Index (broad types) = chance-corrected, label-independent similarity between the two partitions into the three broad types. Adjusted Rand Index (detailed clusters) = the same index applied to the full detailed cluster solutions. The complete-case solution comprised the same number of detailed clusters as the main solution in age groups 1–3 (7, 6, and 8 respectively) and one fewer in age group 4 (5 versus 6). High concordance in the two younger age groups indicates that the imputation of a single missing wave did not alter the typology. The lower concordance in the two older age groups reflects the combined effect of the substantially smaller complete-case sample and the re-estimation of the cluster structure on that sample, rather than the imputation alone.
