# Supplementary material for: Sex and educational inequalities across functional ability trajectories in Chile: A sequence analysis of a 17-year national panel
Source: PLoS One. 2026 Nov 3;21(11):e0361826. doi: 10.1371/journal.pone.0361826 (PMC13730331; doi:10.1371/journal.pone.0361826)
Supplement: Supplementary file 2 [file pone.0361826.s002.docx]

|  | **Age group 1** | | | **Age group 2** | | | **Age group 3** | | | **Age group 4** | | |
| --- | --- | --- | --- | --- | --- | --- | --- | --- | --- | --- | --- | --- |
|  | **Study sample**  **(N=743)** | **Sample including**  **all missing observations**  **(N=1,739)** | **Sample excluding deceased individuals**  **(N=654)** | **Study sample**  **(N=711)** | **Sample including**  **all missing observations**  **(N=1,459)** | **Sample excluding deceased individuals**  **(N=581)** | **Study sample**  **(N=657)** | **Sample including**  **all missing observations**  **(N=1,206)** | **Sample excluding deceased individuals**  **(N=424)** | **Study sample**  **(N=570)** | **Sample including**  **all missing observations**  **(N=1,030)** | **Sample excluding deceased individuals**  **(N=235)** |
| **Sex (%)** | | | | |  |  |  |  |  |  |  |  |
| Women | 54.6 | 51.1 | 56.9 | 55.3 | 50.8 | 58.3 | 49.8 | 47.9 | 53.1 | 46.3 | 47.7 | 59.1 |
| Men | 45.4 | 48.9 | 43.1 | 44.7 | 49.2 | 41.7 | 50.2 | 52.1 | 46.9 | 53.7 | 52.3 | 40.9 |
| **Educational level (%)** | | | | |  |  |  |  |  |  |  |  |
| Primary education or none | 44.3 | 39.3 | 42.2 | 50.1 | 46.6 | 49.1 | 55.1 | 51.7 | 49.8 | 67.0 | 62.7 | 68.1 |
| Secondary education or more | 55.7 | 60.7 | 57.8 | 49.9 | 53.4 | 50.9 | 44.9 | 48.3 | 50.2 | 33.0 | 37.3 | 31.9 |
| **Functional limitation status in baseline year (%)** | | | | | | | | | | | | |
| No limitations | 95.2 | 96.7 | 96.3 | 95.9 | 95.8 | 96.4 | 91.9 | 92.7 | 93.4 | 89.0 | 91.4 | 91.5 |
| Low limitations | 2.6 | 2.0 | 2.1 | 2.7 | 2.7 | 2.9 | 4.0 | 4.2 | 3.5 | 5.6 | 4.4 | 3.8 |
| Middle limitations | 0.5 | 0.3 | 0.5 | 0.1 | 0.2 | 0.0 | 0.3 | 0.2 | 0.2 | 0.7 | 0.6 | 0.4 |
| High limitations | 1.7 | 1.0 | 1.1 | 1.3 | 1.2 | 0.7 | 3.8 | 2.9 | 2.8 | 4.7 | 3.6 | 4.3 |

**S2 Table.** Comparison of key variables in the study sample, a sample with missing observations, and a sample excluding deceased individuals
