# Supplementary material for: Sex and educational inequalities across functional ability trajectories in Chile: A sequence analysis of a 17-year national panel
Source: PLoS One. 2026 Nov 3;21(11):e0361826. doi: 10.1371/journal.pone.0361826 (PMC13730331; doi:10.1371/journal.pone.0361826)
Supplement: Supplementary file 3 [file pone.0361826.s003.docx]

**S3 Appendix.** Sensitivity Analysis 2

As a sensitivity analysis, we re-estimated trajectories using the number of reported limitations instead of the hierarchical state variable (see Supplementary Figure 2). Because both operationalizations partition the same individuals within each baseline age group, we quantified their concordance by cross-classifying each individual’s membership in the three broad trajectory types (see Supplementary Table 3). Concordance was very high in the two younger age groups: 96.0% of individuals in the 46–50 group and 98.0% in the 51–55 group were assigned to the same broad type (Cohen’s κ = 0.93 and 0.96, adjusted Rand index for the broad types = 0.93 in both cases), and the prevalence of the three broad types differed by no more than 4.1 percentage points across operationalizations. Concordance remained substantial in the 56–60 group (agreement = 83.9%, Cohen’s κ = 0.69, adjusted Rand index = 0.57). In the oldest group (61–65), agreement was more moderate (57.4%, Cohen’s κ = 0.26, adjusted Rand index = 0.26): although the same three broad types were identifiable, the number-of-limitations solution reorganized the boundaries between preserved functioning before death and decline-related trajectories, which in this cohort involve a higher concentration of limitation and mortality states. Overall, while the exact number and labelling of detailed clusters differed slightly between operationalizations, and concordance decreased with baseline age, the broad three-type structure and the age-graded shift from sustained stability toward death-related trajectories were preserved across operationalizations.

| **Trajectory Type** | **Age group 1**  (46-50/62-66)  N=743 | **Age group 2**  (51-55/67-71)  N=711 | **Age group 3**  (56-60/72-76)  N=657 | **Age group 4**  (61-65/77-81)  N=570 |
| --- | --- | --- | --- | --- |
|  | **Mostly Non-Declining Trajectories** | | | |
|  | 52.8% | 42.1% | 28.3% | 12.8% |
| Stable good |  |  |  |  |
|  | 6.3% | 9.0% | 6.2% | 11.4% |
| Good before mid-period death |  |  |  |  |
|  |  |  | 27.1% | 33.7% |
| Good before late death |  |  |  |  |
|  | **Progressive-Declining Trajectories** | | | |
|  | 8.6% |  |  |  |
| Late low decline |  |  |  |  |
|  |  |  |  | 12.1% |
| Late low decline before late death |  |  |  |  |
|  |  |  | 16.1% |  |
| Progressive severe decline |  |  |  |  |
|  | 5.0% | 7.0% | 9.4% | 20.5% |
| Progressive decline before early death |  |  |  |  |
|  |  |  | 8.5% |  |
| Progressive decline before late death |  |  |  |  |
|  |  |  |  |  |

**Figure S3.1**. Functional ability trajectory types across four age groups using number of functional limitations

**Figure S3.1**. Functional ability trajectory types across four age groups using number of functional limitations (continued)

| **Trajectory Type** | **Equivocal-Declining Trajectories** | | | |
| --- | --- | --- | --- | --- |
|  | 5.1% |  | 4.3% |  |
| Early low decline |  |  |  |  |
|  | 8.2% | 5.1% |  | 9.5% |
| Early severe decline |  |  |  |  |
|  | 8.3% | 15.6% |  |  |
| Late severe decline |  |  |  |  |
|  | 5.7% | 21.2% |  |  |
| Equivocal recovery |  |  |  |  |
|  |  |  |  |  |

**Table S3.1.** Concordance between the main trajectory typology (hierarchical functional ability state variable) and the sensitivity-analysis typology (number of reported limitations), by baseline age group

| **Baseline age group** | **N** | **Agreement on broad trajectory types (%)** | **Cohen’s κ (broad trajectory types)** | **Adjusted Rand Index (broad trajectory types)** | **Adjusted Rand Index (detailed trajectory types)** | **Maximum absolute difference in broad trajectory type prevalence (percentage points)** |
| --- | --- | --- | --- | --- | --- | --- |
| Age group 1 | 743 | 96.0 | 0.93 | 0.93 | 0.91 | 4.1 |
| Age group 2 | 711 | 98.0 | 0.96 | 0.93 | 0.79 | 1.7 |
| Age group 3 | 657 | 83.9 | 0.69 | 0.57 | 0.61 | 4.9 |
| Age group 4 | 570 | 57.4 | 0.26 | 0.26 | 0.49 | 8.4 |

Notes: This table reports the concordance between two operationalizations of functional ability used to reconstruct long-term trajectories: the hierarchical five-state variable used in the main analysis (no, low, middle, high limitation, and deceased) and the number of reported limitations used in the sensitivity analysis. Both solutions partition the same individuals within each baseline age group, which allows their cluster memberships to be cross-classified directly.

N is the number of individuals in the baseline age group. Agreement on broad types (%) is the percentage of individuals assigned to the same broad trajectory type (mostly non-declining, progressive-declining, or equivocal-declining) under both operationalizations. It is the proportion of cases on the diagonal of the 3×3 cross-classification of broad-type membership. Higher values indicate greater concordance. Cohen’s κ (broad) is the chance-corrected agreement on the three broad types. Unlike the raw percentage, κ discounts the agreement that would be expected by chance given the marginal distribution of the broad types. Values near 1 indicate near-perfect agreement, values near 0 indicate agreement no better than chance (κ ≈ 0.41–0.60 is conventionally interpreted as moderate, 0.61–0.80 as substantial, and >0.80 as almost perfect). Adjusted Rand Index measures the similarity between the two partitions of individuals into the three broad trajectory types and the detailed trajectory types, corrected for chance. Unlike percentage agreement and κ, the Rand index does not require the two solutions to use the same labels, because it compares whether pairs of individuals are grouped together or apart under both solutions. Values near 1 indicate near-identical partitions, values near 0 indicate similarity no greater than expected by chance. Maximum absolute difference in broad trajectory type prevalence is the largest absolute difference, in percentage points, between the two operationalizations in the prevalence of any of the three broad trajectory types within the age group. It summarizes how much the aggregate composition of the typology shifts when functional ability is operationalized differently. Smaller values indicate more stable aggregate prevalence.
