# Supplementary material for: Sex and educational inequalities across functional ability trajectories in Chile: A sequence analysis of a 17-year national panel
Source: PLoS One. 2026 Nov 3;21(11):e0361826. doi: 10.1371/journal.pone.0361826 (PMC13730331; doi:10.1371/journal.pone.0361826)
Supplement: Supplementary file 4 [file pone.0361826.s005.docx]

**Response Letter**

Dear Academic Editor and Reviewer,

We sincerely thank you for the careful and constructive evaluation of our manuscript, now titled “Sex and Educational Inequalities Across Functional Ability Trajectories in Chile: A Sequence Analysis of a 17-Year National Panel”. We are grateful for the opportunity to revise and resubmit the manuscript.

We have undertaken a major revision in response to the comments. The most important change concerns the analytical strategy. In the previous version, trajectory clusters were estimated separately within sex and educational subgroups, which limited the comparability of the central claims.

In the revised manuscript, trajectory typologies are now estimated within each baseline age group using the full pooled age-group-specific sample, and sex and educational differences are examined within these shared trajectory categories. This revised strategy is stated in the Abstract, Materials and Methods, Results, Discussion, and Conclusion.

We also rewrote the Introduction to articulate the research gaps more clearly, strengthened the positioning of the study within the functional ability trajectory literature, expanded the description of the data source and observation period, clarified the handling of missing data and sensitivity analyses, reorganized the Results section around three comparable trajectory types, and revised the Discussion and Conclusion to better distinguish empirical description from interpretation.

Below, we provide a point-by-point response to each comment.

Yours sincerely,

The authors

**Academic Editor**

**1.** The manuscript would be strengthened by clearer reporting of the study characteristics and extent of missing data, ideally in Table 1 or a supplementary table, so that readers can better assess the amount of information addressed through imputation.

Response: We thank the Academic Editor for this suggestion. We revised the Materials and Methods section to provide clearer information about the study characteristics, sample selection, and missing-data criteria. The revised manuscript now states that the study used six observation points from the Chilean Social Protection Survey covering the period 2004–2020, and that the analytic sample included 2,681 individuals aged 46–65 at baseline, contributing 16,086 person-wave observations.

We also clarified that respondents were retained if they had no more than one missing functional ability observation across the six waves. For respondents with one missing functional ability observation, missing states were imputed using multiple imputation by chained equations with predictive mean matching, incorporating age, sex, educational attainment, and adjacent functional ability states as predictors.

These revisions appear in the Materials and Methods section, especially in the subsection “Data source and study design”, where we now describe the data source, observation period, analytic sample, age-group structure, missing-data criterion, and imputation procedure in a more explicit way. The revised Table 1 also reports descriptive characteristics of the analytic sample by baseline age group, including sex, educational attainment, baseline functional ability status, and functional ability status in 2020. In addition, Supplementary Table 1 reports sensitivity analyses comparing the study sample with alternative samples, including the broader sample with missing observations and the sample excluding deceased individuals.

**2.** In addition, a sensitivity analysis examining the impact of the imputation approach would be valuable, as this would help demonstrate the robustness of the findings to assumptions made about missing data.

Response: We thank the Academic Editor for this recommendation. To directly assess the robustness of our findings to the imputation strategy, we conducted an additional sensitivity analysis in which we re-estimated the sequence and cluster analysis on the subsample of individuals with fully complete functional ability information across all six waves, excluding all imputed observations. Because each complete-case individual carries both an original (imputed-sample) and a complete-case trajectory assignment, we cross-classified the two at the individual level and computed the percentage agreement, Cohen’s κ, and the adjusted Rand index for each baseline age group. The results are reported in Supplementary Table 2, and the complete-case typology is shown in Supplementary Figure 1.

In the two younger age groups, the two solutions assigned 100% of individuals to the same broad trajectory type (Cohen’s κ = 1.00), indicating that the imputation of a single missing wave did not alter the typology. In the two older age groups, concordance was lower (agreement of 60.6% and 75.9%). We interpret this difference cautiously. Because mortality is high in these cohorts, the complete-case restriction removed a large share of the sample, and the cluster structure was therefore re-estimated on a substantially smaller and differently composed subsample. The reduced concordance thus reflects the combined effect of sample-size reduction and cluster re-estimation rather than the imputation procedure alone. Importantly, the same three broad trajectory types remained identifiable in all four age groups (Supplementary Figure 1).

In addition to this analysis, and to further probe the robustness of the trajectory typology, we conducted a second sensitivity analysis in which functional ability was operationalized using the number of reported limitations instead of the hierarchical state variable. Cross-classifying each individual's broad-type membership across the two operationalizations showed very high concordance in the younger age groups (agreement of 96.0% and 98.0%; Cohen’s κ = 0.93 and 0.96) and lower concordance in the oldest group, consistent with the higher concentration of limitation and mortality states in that cohort. The broad three-type structure and the age-graded shift from sustained stability toward death-related trajectories were nonetheless preserved across operationalizations. This analysis is reported in Supplementary Table 3 and Supplementary Figure 2.

We have added a description of both sensitivity analyses to the Materials and Methods section (Data source and study design, and Functional ability measure subsections, respectively) and report the full results in the revised Supplementary Material.

**Reviewer**

**1.** This manuscript uses a rich 17-year Chilean panel dataset and applies sequence analysis in a technically manner to explore functional ability trajectories. The use of optimal matching, multiple clustering quality criteria, and clear visualizations represent important methodological strengths. The topic is relevant for life-course and aging research, and the dataset provides a opportunity to examine long-term functional dynamics in an understudied context. However, several important conceptual and methodological issues limit the validity of the manuscript’s central claims.

Response: We sincerely thank the Reviewer for recognizing the strengths of the dataset, the relevance of the topic, and the methodological value of the sequence-analysis approach. We also appreciate the Reviewer’s identification of conceptual and methodological issues that limited the previous version.

In response, we substantially revised the manuscript. The revised version now includes a clearer research gap, stronger positioning within the literature on functional ability trajectories, a corrected analytical strategy based on pooled age-group-specific trajectory typologies, a rewritten Results section organized around comparable trajectory types, and a revised Discussion that interprets the findings in relation to functional aging, life-course inequality, and methodological comparability.

These changes are reflected throughout the manuscript. The Abstract has been rewritten to summarize the revised analytical strategy and main findings. The Introduction now clarifies the research gaps and contribution. The Materials and Methods section explains the pooled age-group-specific clustering strategy and the treatment of missing data. The Results section is reorganized around three main trajectory types, and the Discussion and Conclusion now interpret the findings while emphasizing the methodological contribution of comparing sex and educational groups within shared trajectory categories.

**2.** The study performs a technically sound and sophisticated sequence analysis; however, the way trajectory clusters are constructed separately within each stratified subgroup (by sex, education, and age) makes the resulting trajectories non-comparable across groups, which undermines the central comparative claims of the manuscript.

Response: We agree with this comment. This was the central methodological limitation of the previous version. In the original manuscript, clusters were estimated separately within sex and educational subgroups, which meant that trajectories with similar labels were not necessarily equivalent empirical constructs across groups.

We have corrected this issue in the revised manuscript. Trajectory clusters are now estimated within each baseline age group using the full pooled age-group-specific sample. After creating this common typology for each age group, we compare the sex and educational composition of the resulting shared trajectory types. This ensures that comparisons across women and men, and across educational groups, refer to common trajectory categories within each baseline age group.

This revised strategy is introduced in the Abstract and developed in the Materials and Methods section, especially in the subsections “Sequence analysis and trajectory classification” and “Analytical strategy”. The Results section then applies this strategy by presenting trajectory types estimated within pooled age-group samples and by comparing sex and educational composition within those shared types. The Discussion and Conclusion explicitly interpret this methodological change as a way to provide a comparable account of sex and educational differences in long-term functional ability trajectories.

We have also applied the same comparability logic to clarify the status of comparisons made across baseline age groups. Within each age group, the pooled estimation guarantees that sex and educational groups are compared within statistically shared trajectory categories. Across age groups, however, the grouping of the age-group-specific cluster solutions into three broad types (mostly non-declining, progressive-declining, and equivocal-declining) is an interpretive harmonization based on the shape, ordering, and timing of the detailed trajectories, rather than a single jointly estimated cross-age cluster solution. We now state this explicitly so that within-age comparisons (statistically grounded) and cross-age comparisons (descriptive) are not conflated. This clarification is reflected in the Results section, where the three broad types are introduced as a harmonization of the detailed age-group-specific trajectories, and in the Limitations subsection of the Discussion, where we note that comparisons across baseline age groups should be read as descriptive rather than as contrasts between strictly equivalent constructs.

**3.** The introduction lacks a clearly articulated research gap and does not sufficiently position the study within the existing literature on functional ability trajectories and sequence analysis methodology. Forever, the theoretical background and anchoring the present research in the current state of empirical results of other studies are somewhat weak. I should consider identifying, reviewing Relevant literature on the analyzed topic and strengthen this part of the paper.

Response: We have substantially rewritten the Introduction to address this concern. The revised Introduction now identifies two explicit research gaps. First, most evidence on long-term functional ability trajectories comes from the United States, Europe, and parts of Asia, while evidence from Latin America remains more limited. Second, the revised Introduction explains that comparisons across sex and educational groups require trajectory typologies constructed in a way that allows valid comparison across groups.

We also strengthened the theoretical and empirical framing. The revised Introduction discusses the shift from static disability measures toward longitudinal functional ability trajectories, the relevance of life-course and bridging approaches, previous international evidence on disability and functional ability trajectories, and prior Chilean evidence using the EPS. The revised version also clarifies why education is relevant as a marker of cumulative life-course resources.

These changes appear throughout the revised Introduction. The opening paragraphs now frame functional ability as a dynamic process and situate the study within longitudinal disability and functional ability research. The subsection “Research gaps” explicitly identifies the empirical and methodological gaps addressed by the study. The final paragraphs of the Introduction then clarify how the present study extends previous Chilean research and state the research question guiding the analysis.

**4.** Building methodological section, it should be provided the source of the data as the reference in the paper. Also, the reason the author decided to analyze should be explained: they thought we jar very old from 2004 to 2010.

Response: We revised the Materials and Methods section to provide a clearer description of the data source and the rationale for the analytic period and baseline age range. The revised manuscript now identifies the *Encuesta de Protección Social* (Social Protection Survey, EPS) as a nationally representative Chilean longitudinal survey and includes a reference to the official EPS data source.

We also explain why the 2004–2020 observation window was used. The period covers all the surveys available to date. This period provides six observation points with functional ability information and allows us to examine functional ability changes over 17 years, from midlife into older age. We further clarify why individuals aged 46–65 in 2004 were selected: this baseline age range allows the analysis to begin before most respondents reach older ages while still observing transitions into later life. Respondents were then divided into four five-year baseline age groups: 46–50, 51–55, 56–60, and 61–65.

These revisions are concentrated in the Materials and Methods section, particularly in the subsection “Data source and study design”. In that subsection, we now identify the EPS as the data source, cite the official EPS data reference, justify the 2004–2020 observation window, explain the selection of individuals aged 46–65 at baseline, and describe the rationale for analyzing four five-year baseline age groups separately.

**5.** By generating different cluster solutions within each stratified subgroup, the study creates group-specific trajectory types and then compares their prevalence as if they were equivalent constructs, which constitutes a methodological inconsistency.

Response: We agree with the Reviewer. This methodological inconsistency has been corrected. In the revised manuscript, we no longer generate sex-specific or education-specific cluster solutions. Instead, for each baseline age group, we estimate one pooled trajectory typology using the full age-group-specific sample. We then compare the sex and educational composition of the resulting shared trajectory types.

This revision changes the interpretation of the Results. The manuscript no longer compares the prevalence of non-equivalent clusters across separately stratified subgroup solutions. It now compares the distribution of sex and educational groups within common trajectory categories.

This correction is described in the Materials and Methods section, where we explain the pooled age-group-specific clustering strategy. The Results section then follows this revised design by presenting trajectory types defined before sex and education are compared. The subsections “Sex differences in trajectory types across age groups” and “Educational differences in trajectory types across age groups” report the composition of shared trajectory types by sex and education. Tables 2 and 3 support this analysis by reporting the sex and educational composition of main and detailed trajectory types within each age group. The Discussion explains why this revised strategy strengthens the validity of the comparative claims.

We would like to clarify one remaining aspect of comparability that follows from the Reviewer’s observation. The pooled estimation resolves the comparability problem for sex and educational groups within each baseline age group, because these groups are now compared within statistically equivalent trajectory categories. The grouping of the detailed, age-group-specific clusters into three broad types, however, is an interpretive harmonization rather than a jointly estimated typology spanning all age groups. We have made this distinction explicit to avoid implying that the underlying detailed clusters are statistically equivalent across age groups. Accordingly, the Results section now presents the three broad types as an interpretive grouping of the age-group-specific trajectories, intended to support cross-age description, and the Limitations subsection notes that comparisons across baseline age groups are descriptive in nature, while comparisons by sex and education within each age group rest on the shared, pooled trajectory categories.

**6.** When constructing the text, the ideas should be consolidated in Francis and paragraphs, not on short sentences and paragraph with 1 1/2 or two lines. Moreover, should be avoided structuring the text in very short bullet points, like on lines 208-212, 222-225, 231-234,

Response: We revised the manuscript’s writing style to address this comment. The previous Results section included short bullet-style descriptions of trajectory subtypes. In the revised manuscript, these descriptions have been rewritten as continuous prose. We also consolidated short paragraphs throughout the Introduction, Materials and Methods, Results, and Discussion sections to improve flow and readability.

This revision is especially visible in the Results section. The subsection “Functional ability trajectories across age groups” now describes the detailed trajectories in integrated paragraphs rather than short bullet points. The sex and education subsections also present group differences in narrative prose. The Discussion has been reorganized into interpretive paragraphs that synthesize the findings and reduce unnecessary repetition with the Results section.

**7.** Considering above consideration, the paper should undergo a major revision before being considered for publication.

Response: We agree that a major revision was necessary. The manuscript has been substantially revised in response to the Reviewer’s comments. The revised version includes a corrected analytical strategy, a clearer research gap, a stronger methodological explanation, updated figures and tables, a rewritten Results section based on pooled age-group-specific trajectory typologies, and a revised Discussion and Conclusion.

These changes affect the manuscript as a whole. The revision corrects the methodological inconsistency identified by the Reviewer, strengthens the Introduction and Methods, reorganizes the Results around comparable trajectory categories, and clarifies the interpretation of sex and educational differences in the Discussion and Conclusion. We believe these changes directly address the Reviewer’s concerns and substantially improve the manuscript’s methodological validity, clarity, and contribution.
