# Supplementary material for: Sex and educational inequalities across functional ability trajectories in Chile: A sequence analysis of a 17-year national panel
Source: PLoS One. 2026 Nov 3;21(11):e0361826. doi: 10.1371/journal.pone.0361826 (PMC13730331; doi:10.1371/journal.pone.0361826)
Supplement: Supplementary file 5 [file pone.0361826.s006.docx]

**Response Letter**

Dear Academic Editor and Reviewer,

We sincerely thank you for the opportunity to revise and resubmit our manuscript, “Sex and Educational Inequalities Across Functional Ability Trajectories in Chile: A Sequence Analysis of a 17-Year National Panel”. We are particularly grateful to the Reviewer for a set of comments that were detailed, constructive, and closely informed by the data source we use. Several of them identified issues that we had not documented adequately and that we have now addressed throughout the manuscript.

The most important change concerns the interpretation of our central findings. Following the Reviewer’s eighth comment, we now reference the sex and educational composition of every trajectory type to the marginal composition of its baseline age group, both in Tables 2 and 3 and in the Results and Discussion. This has materially improved the analysis, as it allows us to distinguish compositional differences that reverse the aggregate distribution of an age group from those that reproduce it, and it revealed that one of our previous claims required qualification. The apparent concentration of respondents with primary education or less in equivocal-declining trajectories in the oldest age group (65.7%) in fact falls slightly below that age group’s marginal (67.0%), and all three trajectory types in that age group track the marginal educational distribution within 7.2 percentage points, which now provides a substantive account of why the chi-squared test is non-significant there.

Beyond this, we have made the following changes. The Introduction now specifies the mechanisms through which individual and social factors shape functional decline in high-income settings. The Research gaps subsection articulates why the Chilean configuration of rapid demographic aging, recent educational expansion, a historically deregulated labor market, and a segmented health and long-term care system leads to specific expectations about how functional trajectories unfold, and the Discussion returns to these expectations. The Materials and Methods section now documents the selection of EPS waves explicitly, including the reasons for excluding the 2002, 2012, and 2024 waves, and acknowledges the disruption of the 2020 fieldwork. We have clarified the representativeness of the analytic sample, stated and justified the decision not to apply survey weights, and removed an undefined acronym. Six new references have been added and the reference list has been renumbered accordingly.

Below we respond to each comment in turn, indicating where the corresponding changes appear.

Yours sincerely,

The authors.

**REVIEWER**

**Comment 1.** It is stated that previous research on high-income countries has shown that individual and social factors shape functional decline, but how this operates is not made explicit (i.e., what do we already know from these contexts?)

Response: We thank the Reviewer for this observation. The previous version listed the factors associated with functional decline without specifying how they operate. We have rewritten the third paragraph of the Introduction to describe the mechanisms documented in high-income settings, distinguishing sex-related, socioeconomic, and clinical pathways.

The revised paragraph specifies the configuration underlying sex differences, namely a higher burden of non-fatal disabling conditions, differences in muscle mass and strength, and lifetime differences in paid work and caregiving responsibilities, alongside longer survival among women. It then specifies the intermediate channels through which educational attainment operates, including exposure to physically demanding and hazardous occupations, health literacy and the capacity to navigate health systems, income and material conditions in later life, and health behaviors. It also clarifies the dual role of chronic conditions, multimorbidity, and sedentary behavior, which act both as precipitating events that trigger transitions into limitation and as constraints on the likelihood of recovering function once limitation has occurred, so that advantaged groups more frequently display recovery and less frequently remain in unstable states. We further note that these mechanisms accumulate across the life course, with early-life circumstances and adulthood employment histories shaping functional status decades later, and that the strength and direction of specific associations varies across age groups and cohorts.

Two references on sex differences in health and survival and one on childhood socioeconomic position and later-life functional limitations in Chile have been added to support these mechanisms.

**Comment 2.** It is argued that a main gap is that evidence so far comes mainly from the US, Europe and Asia, and that evidence from Latin America is scarce, even though the region faces rapid population ageing, high inequality and different institutional arrangements. I think the argument could be pushed a little further to say why these particularities make this research gap relevant (e.g., what should we expect to be different from/similar to what we already know from previous research?). This could also be further re-emphasised within the discussion or conclusion sections.

Response: We agree that the argument required further development. The previous version stated that the region differs from the settings where most evidence has been produced without specifying what those differences imply for the phenomenon under study. We have added a new paragraph to the Research Gaps subsection that articulates why the Chilean configuration matters and what it leads us to expect.

Specifically, we now discuss four features and their implications. The compressed pace of demographic aging means that successive cohorts reach later life with markedly different educational and occupational endowments. Rapid educational expansion produces steep cohort gradients in educational attainment, which entangles educational differences with cohort composition and shifts the social meaning of a given educational threshold across baseline age groups. A labor market characterized by weak employment protection and persistent informality concentrates cumulative occupational exposure among lower-educated workers, which plausibly produces steeper educational gradients than in settings with stronger labor regulation. A segmented health system and limited long-term care provision make recovery from an episode of limitation dependent on household resources, which suggests that socioeconomic differences will be expressed in the stability and reversibility of trajectories as much as in their onset. We also state which features we expect to resemble those documented elsewhere, namely the basic heterogeneity of functional pathways.

Following the Reviewer's suggestion, we return to this argument in the Discussion with a dedicated paragraph that assesses our findings against these expectations. First, we note that the concentration of women and of lower-educated respondents in equivocal-declining trajectories is consistent with recovery being conditional on household resources. Second, we observe that the strengthening of the educational gradient in progressive-declining trajectories with baseline age is consistent with cumulative occupational exposure. Finally, we argue that the heterogeneity of the trajectories themselves resembles that reported in higher-income settings, so that the distinctiveness of the Chilean case lies in how social groups are distributed across these pathways rather than in the existence of the pathways themselves. The Conclusion has been adjusted accordingly.

**Comment 3.** EPS waves selection: It is stated that the period 2004-2020 covers all EPS available waves. However, there is also a 2002 wave and a 2024 wave. I assume the former wasn’t used as it is not representative of the adult population but only of those affiliated to the pension system (or perhaps because of the lack of any relevant variable?) and the latter as it was probably not yet available when the analysis was undertaken, but it is worth mentioning whether this is the case.

Response: We thank the Reviewer for noting this, and we confirm that both assumptions are correct. The statement that the study used all available EPS waves was inaccurate and has been removed.

The revised Data source and study design subsection now lists all waves fielded by the EPS (2002, 2004, 2006, 2009, 2012, 2015, 2018, 2020, and 2024) and explains the selection of the analytic window. The 2002 wave was excluded for the two reasons the Reviewer anticipates, that is, questions on functional ability status were incorporated into the survey only from the 2004 version onwards, and the 2002 sample was restricted to individuals affiliated to the pension system, which limits its representativeness of the adult population. The 2024 wave had not been released at the time the analyses were conducted, and we now state that it will be incorporated into future extensions of this work. Additionally, the 2012 wave was also excluded, for reasons of data quality that we detail in our response to Comment 4 below.

**Comment 4.** EPS waves limitations: Some additional issues with the EPS data might be acknowledged (and/or reflect on whether they might have any implications for the analysis). For instance, EPS 2012 data were published with some reservations due to low response rates during fieldwork and the institution responsible for the survey suggests caution when using them for statistical inference. In addition, fieldwork for the EPS 2020 wave had to be suspended during mid-March 2020 due to Covid-19 lockdown measures, resulting in a smaller sample size, and although the survey was continued from September to December of that year, it changed mode to a phone survey.

Response: We are grateful for this observation, which led us to document two data-quality issues that the previous version did not acknowledge.

The revised Data source and study design subsection now includes a dedicated paragraph stating that the 2012 wave was published with reservations by the institution responsible for the survey, due to low response rates during fieldwork and an accompanying recommendation that these data be used with caution for statistical inference, and that it was therefore excluded from the analytic window. The same paragraph describes the disruption of the 2020 fieldwork, which began face-to-face in late 2019, was suspended in March 2020 following the introduction of nationwide lockdown measures, and resumed between September and December of that year by telephone, yielding a smaller achieved sample and a change in survey mode for part of the wave.

We have also added a new limitation (now the third) reflecting on the implications for our analysis. We acknowledge that mode effects on the self-report of difficulties in activities of daily living cannot be ruled out, and that the reduced achieved sample may have introduced additional selectivity at the endpoint of the observation window.

We note three features of our design that limit the leverage of this issue on our conclusions. First, trajectories are constructed from whole sequences of six observations, so that any single wave exerts bounded influence on cluster assignment. Second, the functional ability measure is a coarse hierarchical classification that is less sensitive to fine-grained reporting differences than a continuous score. And third, the sensitivity analysis based on the number of reported limitations reproduced the broad trajectory structure. We state that results concerning the final period should nonetheless be interpreted with this caveat in mind.

**Comment 5.** Table 1 description: When describing Table 1, the acronym FA is used (in line 156). I assume it refers to functional ability, but I think it is not previously defined in the text. FA is also used in line 225 when describing the sequence analysis.

Response: We thank the Reviewer for identifying this inconsistency. The acronym was indeed used without prior definition. Since it appeared only twice, we have replaced both occurrences with the full term “functional ability” rather than introducing an abbreviation, which improves readability.

The affected passages are the description of Table 1 in the Data source and study design subsection and the description of the cluster selection procedure in the Sequence analysis and trajectory classification subsection.

**Comment 6.** Analytical sample: The analytical sample is composed of 2,681 individuals aged 45-64 in 2004 across the 6 EPS waves. Is this sample representative of the population in that age group? (i.e., how might the attrition of the survey be affecting this, given that the analyses may miss people who are only present in some of the waves?).

Response: We thank the Reviewer for raising this point, which we have addressed in two ways.

First, we now state explicitly that the analytic sample is defined by observability of functional ability status across the six waves, and is therefore representative of respondents who remained in the panel rather than of the Chilean population aged 46–65 in 2004.

Second, we have expanded the discussion of Supplementary Table 1, which compares the analytic sample with the broader sample that includes individuals with missing observations. The differences are modest and consistent in direction across age groups. The analytic sample contains between 3.4 and 5.0 percentage points more respondents with primary education or less across the four age groups, a somewhat higher proportion of women in the three younger age groups, and a marginally lower baseline prevalence of no limitations in three of the four age groups. Retention therefore does not appear to have selected the most advantaged or the healthiest respondents, which is reassuring for a study concerned with functional decline. We also note that retaining individuals who died during the observation period, and treating death as a functional ability state, limits one of the principal sources of selective loss in long panels of aging populations.

The second limitation has been expanded accordingly, acknowledging that individuals lost to follow-up for reasons correlated with unobserved health deterioration cannot be recovered and that some selection bias cannot be ruled out.

**Comment 7.** Sampling weights: Does the analysis use any survey sampling weights? This should be stated, and the reason for the choice justified.

Response: We thank the Reviewer for this request. The analyses are unweighted, and the previous version neither stated nor justified this decision. We have now made it explicit in a new paragraph of the Analytical Strategy subsection and provided three reasons.

First, the construction of the typology rests on pairwise dissimilarities between sequences and on hierarchical clustering, procedures into which expansion factors cannot be incorporated in a straightforward manner at the stage where trajectory types are defined. Second, our substantive claims concern the sex and educational composition of shared trajectory categories within each baseline age group, a within-sample comparison for which weighting is less consequential than it would be for national prevalence estimation. Third, previous research using the same survey and a comparable trajectory design reported highly similar results with and without the expansion factor, which is consistent with an accurate sampling strategy and adequate fieldwork in the EPS.

We have also added a new limitation (now the fourth) stating that, because analyses are unweighted, the prevalence figures reported for each trajectory type describe the analytic sample and should not be read as population estimates, while noting that the compositional comparisons supporting our substantive claims are internal to each age group.

**Comment 8.** When presenting/discussing the findings of the sex and educational differences in trajectory types across age groups, it will be interesting to relate them to the respective prevalent differences in sex and educational categories by each group and whether or not it is consistent. For example, the mostly non-declining trajectories had a higher proportion of men in age groups 1, 3, and 4 and the progressive-declining trajectories had a higher proportion of women in those same age groups. Given that age groups 1 and 2 overall have more women than men and age groups 3 and 4 more men than women, the disaggregation by trajectory types may be “reverting” or “reinforcing” some of this aggregate composition. The same applies to the education findings discussion.

Response: We are grateful for this suggestion, which substantially improved the interpretation of our findings and led to the most extensive revision in this round. The previous version reported the composition of each trajectory type without anchoring it to the overall composition of the corresponding baseline age group, so that readers could not assess whether a given composition departed from or reproduced the aggregate distribution.

We have addressed this in four ways.

First, Tables 2 and 3 now include a first row reporting the marginal sex and educational distribution of each baseline age group, so that over- and under-representation can be read directly from the tables. The table notes explain this addition.

Second, the sex subsection now opens with an explicit statement of the marginals and of the convention we adopt, following the Reviewer’s own terms, that is, a departure is described as reversing the aggregate composition when the majority within a trajectory type differs from the majority in the age group, and as reinforcing it when the two coincide. Every composition is then expressed as a departure in percentage points. The male majority in mostly non-declining trajectories reverses the aggregate composition in age groups 1 and 2 (departures of 6.2 and 5.6 percentage points from female-majority marginals) and reinforces it in age groups 3 and 4 (4.5 and 8.6 percentage points). The female majority in equivocal-declining trajectories reinforces the aggregate composition in age groups 1 and 2 (10.2 and 8.0 percentage points) and reverses it in age group 4, where it constitutes the largest departure in the table at 18.4 percentage points. In age group 3 this type closely reproduces a nearly balanced marginal. Among progressive-declining trajectories, three of the four departures reverse the aggregate composition.

Third, the educational subsection follows the same logic. Mostly non-declining trajectories fall below the marginal proportion with primary education or less in all four age groups, by 4.2, 5.8, 4.7, and 3.1 percentage points. Progressive-declining trajectories move from 3.8 percentage points below the marginal in age group 1 to 3.9, 5.5, and 7.2 percentage points above it in age groups 2, 3, and 4, so that the educational gradient strengthens with baseline age relative to the composition of each age group and not only in absolute terms. Equivocal-declining trajectories exceed the marginal by 8.9, 5.8, and 10.7 percentage points in the three younger age groups.

A qualification of our earlier claim. Applying this framework revealed that one statement in the previous version required correction. We had described equivocal-declining trajectories as predominantly composed of lower-educated respondents in all four age groups, citing 65.7% in age group 4. Referenced to that age group’s marginal of 67.0%, this composition in fact falls 1.3 percentage points below the aggregate distribution and therefore does not indicate over-representation. We now state this explicitly, and we note that all three trajectory types in age group 4 fall within 7.2 percentage points of the marginal educational distribution, which provides a substantive interpretation of the non-significant chi-squared test in that age group. The Abstract has been amended so that the educational claim is restricted to the three younger age groups.

The Discussion now includes a dedicated paragraph drawing these threads together, and the Conclusion notes that aging research benefits from referencing trajectory composition to the aggregate composition of the population under study.

We thank again the Reviewer for giving us the opportunity to improve our study.
