# Supplementary material for: Sex and educational inequalities across functional ability trajectories in Chile: A sequence analysis of a 17-year national panel
Source: PLoS One. 2026 Nov 3;21(11):e0361826. doi: 10.1371/journal.pone.0361826 (PMC13730331; doi:10.1371/journal.pone.0361826)
Supplement: Peer review history

# Peer review: Decision Letter 0

**DOI:** 10.1371/journal.pone.0361826.r001

**Authors:** Oyelola A. Adegboye

6 Jun 2026

Dear Dr. Villalobos Dintrans,

Thank you for submitting your manuscript to PLOS ONE. After careful consideration, we feel that it has merit but does not fully meet PLOS ONE’s publication criteria as it currently stands. Therefore, we invite you to submit a revised version of the manuscript that addresses the points raised during the review process.

==============================

**ACADEMIC EDITOR:**

==============================

Please submit your revised manuscript by Jul 21 2026 11:59PM. If you will need more time than this to complete your revisions, please reply to this message or contact the journal office at plosone@plos.org. When you're ready to submit your revision, log on to https://www.editorialmanager.com/pone/ and select the 'Submissions Needing Revision' folder to locate your manuscript file.

- A letter that responds to each point raised by the academic editor and reviewer(s). You should upload this letter as a separate file labeled 'Response to Reviewers'.
- A marked-up copy of your manuscript that highlights changes made to the original version. You should upload this as a separate file labeled 'Revised Manuscript with Track Changes'.
- An unmarked version of your revised paper without tracked changes. You should upload this as a separate file labeled 'Manuscript'.

If you would like to make changes to your financial disclosure, please include your updated statement in your cover letter. Guidelines for resubmitting your figure files are available below the reviewer comments at the end of this letter.

If applicable, we recommend that you deposit your laboratory protocols in protocols.io to enhance the reproducibility of your results. Protocols.io assigns your protocol its own identifier (DOI) so that it can be cited independently in the future. For instructions see: https://journals.plos.org/plosone/s/submission-guidelines#loc-laboratory-protocols. Additionally, PLOS ONE offers an option for publishing peer-reviewed Lab Protocol articles, which describe protocols hosted on protocols.io. Read more information on sharing protocols at https://plos.org/protocols?utm_medium=editorial-email&utm_source=authorletters&utm_campaign=protocols.

As the corresponding author, your ORCID iD is verified in the submission system and will appear in the published article. PLOS supports the use of ORCID, and we encourage all coauthors to register for an ORCID iD and use it as well. Please encourage your coauthors to verify their ORCID iD within the submission system before final acceptance, as unverified ORCID iDs will not appear in the published article. *Only*  the individual author can complete the verification step; PLOS staff *cannot*  verify ORCID iDs on behalf of authors.

We look forward to receiving your revised manuscript.

Kind regards,

Oyelola A. Adegboye, PhD

Academic Editor

PLOS One

**Journal Requirements:**

When submitting your revision, we need you to address these additional requirements.

1. Please ensure that your manuscript meets PLOS ONE's style requirements, including those for file naming. The PLOS ONE style templates can be found at

https://journals.plos.org/plosone/s/file?id=wjVg/PLOSOne_formatting_sample_main_body.pdf and

https://journals.plos.org/plosone/s/file?id=ba62/PLOSOne_formatting_sample_title_authors_affiliations.pdf

2. Please note that PLOS One has specific guidelines on code sharing for submissions in which author-generated code underpins the findings in the manuscript. In these cases, we expect all author-generated code to be made available without restrictions upon publication of the work. Please review our guidelines at https://journals.plos.org/plosone/s/materials-and-software-sharing#loc-sharing-code and ensure that your code is shared in a way that follows best practice and facilitates reproducibility and reuse.

3. Thank you for stating the following financial disclosure:

“This work was supported by the following grants of the Agencia Nacional de Investigación y Desarrollo (ANID), Chile: ANID/ FONDECYT/ REGULAR N°1220080; ANID/ FONDAP Nº15130009; ANID/ Millennium Science Initiative Program ICS2019_024.”

Please state what role the funders took in the study.  If the funders had no role, please state: "The funders had no role in study design, data collection and analysis, decision to publish, or preparation of the manuscript."

If this statement is not correct you must amend it as needed.

Please include this amended Role of Funder statement in your cover letter; we will change the online submission form on your behalf.

4. In the online submission form you indicate that your data is not available for proprietary reasons and have provided a contact point for accessing this data. Please note that your current contact point is a co-author on this manuscript. According to our Data Policy, the contact point must not be an author on the manuscript and must be an institutional contact, ideally not an individual. Please revise your data statement to a non-author institutional point of contact, such as a data access or ethics committee, and send this to us via return email. Please also include contact information for the third party organization, and please include the full citation of where the data can be found.

5. Please include captions for your Supporting Information files at the end of your manuscript, and update any in-text citations to match accordingly. Please see our Supporting Information guidelines for more information: http://journals.plos.org/plosone/s/supporting-information.

6. If the reviewer comments include a recommendation to cite specific previously published works, please review and evaluate these publications to determine whether they are relevant and should be cited. There is no requirement to cite these works unless the editor has indicated otherwise.

[Note: HTML markup is below. Please do not edit.]

Reviewers' comments:

Reviewer's Responses to Questions

**Comments to the Author**

1. Is the manuscript technically sound, and do the data support the conclusions?

Reviewer #1: Partly

**********

2. Has the statistical analysis been performed appropriately and rigorously?

Reviewer #1: Yes

**********

3. Have the authors made all data underlying the findings in their manuscript fully available?

The PLOS Data policy

Reviewer #1: Yes

**********

4. Is the manuscript presented in an intelligible fashion and written in standard English?

Reviewer #1: Yes

**********

Reviewer #1: This manuscript uses a rich 17-year Chilean panel dataset and applies sequence analysis in a technically manner to explore functional ability trajectories. The use of optimal matching, multiple clustering quality criteria, and clear visualizations represent important methodological strengths. The topic is relevant for life-course and aging research, and the dataset provides a opportunity to examine long-term functional dynamics in an understudied context.

However, several important conceptual and methodological issues limit the validity of the manuscript’s central claims.

The study performs a technically sound and sophisticated sequence analysis; however, the way trajectory clusters are constructed separately within each stratified subgroup (by sex, education, and age) makes the resulting trajectories non-comparable across groups, which undermines the central comparative claims of the manuscript.

The introduction lacks a clearly articulated research gap and does not sufficiently position the study within the existing literature on functional ability trajectories and sequence analysis methodology. Forever, the theoretical background and anchoring the present research in the current state of empirical results of other studies are somewhat weak. I should consider identifying, reviewing Relevant literature on the analyzed topic and strengthen this part of the paper.

Building methodological section, it should be provided the source of the data as the reference in the paper. Also, the reason the author decided to analyze should be explained: they thought we jar very old from 2004 to 2010.

By generating different cluster solutions within each stratified subgroup, the study creates group-specific trajectory types and then compares their prevalence as if they were equivalent constructs, which constitutes a methodological inconsistency.

When constructing the text, the ideas should be consolidated in Francis and paragraphs, not on short sentences and paragraph with 1 1/2 or two lines. Moreover, should be avoided structuring the text in very short bullet points, like on lines 208-212, 222-225, 231-234,

Considering above consideration, the paper should undergo a major revision before being considered for publication.

**********

what does this mean?). If published, this will include your full peer review and any attached files.

If you choose “no”, your identity will remain anonymous but your review may still be made public.

**Do you want your identity to be public for this peer review?**  For information about this choice, including consent withdrawal, please see our Privacy Policy

Reviewer #1: No

**********

[NOTE: If reviewer comments were submitted as an attachment file, they will be attached to this email and accessible via the submission site. Please log into your account, locate the manuscript record, and check for the action link "View Attachments". If this link does not appear, there are no attachment files.]

To ensure your figures meet our technical requirements, please review our figure guidelines: https://journals.plos.org/plosone/s/figures

You may also use PLOS’s free figure tool, NAAS, to help you prepare publication quality figures: https://journals.plos.org/plosone/s/figures#loc-tools-for-figure-preparation.

NAAS will assess whether your figures meet our technical requirements by comparing each figure against our figure specifications.

# Author comment: Author response to Decision Letter 1

**DOI:** 10.1371/journal.pone.0361826.r002

7 Jul 2026

Response Letter

Dear Academic Editor and Reviewer,

We sincerely thank you for the careful and constructive evaluation of our manuscript, now titled “Sex and Educational Inequalities Across Functional Ability Trajectories in Chile: A Sequence Analysis of a 17-Year National Panel”. We are grateful for the opportunity to revise and resubmit the manuscript.

We have undertaken a major revision in response to the comments. The most important change concerns the analytical strategy. In the previous version, trajectory clusters were estimated separately within sex and educational subgroups, which limited the comparability of the central claims.

In the revised manuscript, trajectory typologies are now estimated within each baseline age group using the full pooled age-group-specific sample, and sex and educational differences are examined within these shared trajectory categories. This revised strategy is stated in the Abstract, Materials and Methods, Results, Discussion, and Conclusion.

We also rewrote the Introduction to articulate the research gaps more clearly, strengthened the positioning of the study within the functional ability trajectory literature, expanded the description of the data source and observation period, clarified the handling of missing data and sensitivity analyses, reorganized the Results section around three comparable trajectory types, and revised the Discussion and Conclusion to better distinguish empirical description from interpretation.

Below, we provide a point-by-point response to each comment.

Yours sincerely,

The authors

Academic Editor

1. The manuscript would be strengthened by clearer reporting of the study characteristics and extent of missing data, ideally in Table 1 or a supplementary table, so that readers can better assess the amount of information addressed through imputation.

Response: We thank the Academic Editor for this suggestion. We revised the Materials and Methods section to provide clearer information about the study characteristics, sample selection, and missing-data criteria. The revised manuscript now states that the study used six observation points from the Chilean Social Protection Survey covering the period 2004–2020, and that the analytic sample included 2,681 individuals aged 46–65 at baseline, contributing 16,086 person-wave observations.

We also clarified that respondents were retained if they had no more than one missing functional ability observation across the six waves. For respondents with one missing functional ability observation, missing states were imputed using multiple imputation by chained equations with predictive mean matching, incorporating age, sex, educational attainment, and adjacent functional ability states as predictors.

These revisions appear in the Materials and Methods section, especially in the subsection “Data source and study design”, where we now describe the data source, observation period, analytic sample, age-group structure, missing-data criterion, and imputation procedure in a more explicit way. The revised Table 1 also reports descriptive characteristics of the analytic sample by baseline age group, including sex, educational attainment, baseline functional ability status, and functional ability status in 2020. In addition, Supplementary Table 1 reports sensitivity analyses comparing the study sample with alternative samples, including the broader sample with missing observations and the sample excluding deceased individuals.

2. In addition, a sensitivity analysis examining the impact of the imputation approach would be valuable, as this would help demonstrate the robustness of the findings to assumptions made about missing data.

Response: We thank the Academic Editor for this recommendation. To directly assess the robustness of our findings to the imputation strategy, we conducted an additional sensitivity analysis in which we re-estimated the sequence and cluster analysis on the subsample of individuals with fully complete functional ability information across all six waves, excluding all imputed observations. Because each complete-case individual carries both an original (imputed-sample) and a complete-case trajectory assignment, we cross-classified the two at the individual level and computed the percentage agreement, Cohen’s κ, and the adjusted Rand index for each baseline age group. The results are reported in Supplementary Table 2, and the complete-case typology is shown in Supplementary Figure 1.

In the two younger age groups, the two solutions assigned 100% of individuals to the same broad trajectory type (Cohen’s κ = 1.00), indicating that the imputation of a single missing wave did not alter the typology. In the two older age groups, concordance was lower (agreement of 60.6% and 75.9%). We interpret this difference cautiously. Because mortality is high in these cohorts, the complete-case restriction removed a large share of the sample, and the cluster structure was therefore re-estimated on a substantially smaller and differently composed subsample. The reduced concordance thus reflects the combined effect of sample-size reduction and cluster re-estimation rather than the imputation procedure alone. Importantly, the same three broad trajectory types remained identifiable in all four age groups (Supplementary Figure 1).

In addition to this analysis, and to further probe the robustness of the trajectory typology, we conducted a second sensitivity analysis in which functional ability was operationalized using the number of reported limitations instead of the hierarchical state variable. Cross-classifying each individual's broad-type membership across the two operationalizations showed very high concordance in the younger age groups (agreement of 96.0% and 98.0%; Cohen’s κ = 0.93 and 0.96) and lower concordance in the oldest group, consistent with the higher concentration of limitation and mortality states in that cohort. The broad three-type structure and the age-graded shift from sustained stability toward death-related trajectories were nonetheless preserved across operationalizations. This analysis is reported in Supplementary Table 3 and Supplementary Figure 2.

We have added a description of both sensitivity analyses to the Materials and Methods section (Data source and study design, and Functional ability measure subsections, respectively) and report the full results in the revised Supplementary Material.

Reviewer

1. This manuscript uses a rich 17-year Chilean panel dataset and applies sequence analysis in a technically manner to explore functional ability trajectories. The use of optimal matching, multiple clustering quality criteria, and clear visualizations represent important methodological strengths. The topic is relevant for life-course and aging research, and the dataset provides a opportunity to examine long-term functional dynamics in an understudied context. However, several important conceptual and methodological issues limit the validity of the manuscript’s central claims.

Response: We sincerely thank the Reviewer for recognizing the strengths of the dataset, the relevance of the topic, and the methodological value of the sequence-analysis approach. We also appreciate the Reviewer’s identification of conceptual and methodological issues that limited the previous version.

In response, we substantially revised the manuscript. The revised version now includes a clearer research gap, stronger positioning within the literature on functional ability trajectories, a corrected analytical strategy based on pooled age-group-specific trajectory typologies, a rewritten Results section organized around comparable trajectory types, and a revised Discussion that interprets the findings in relation to functional aging, life-course inequality, and methodological comparability.

These changes are reflected throughout the manuscript. The Abstract has been rewritten to summarize the revised analytical strategy and main findings. The Introduction now clarifies the research gaps and contribution. The Materials and Methods section explains the pooled age-group-specific clustering strategy and the treatment of missing data. The Results section is reorganized around three main trajectory types, and the Discussion and Conclusion now interpret the findings while emphasizing the methodological contribution of comparing sex and educational groups within shared trajectory categories.

2. The study performs a technically sound and sophisticated sequence analysis; however, the way trajectory clusters are constructed separately within each stratified subgroup (by sex, education, and age) makes the resulting trajectories non-comparable across groups, which undermines the central comparative claims of the manuscript.

Response: We agree with this comment. This was the central methodological limitation of the previous version. In the original manuscript, clusters were estimated separately within sex and educational subgroups, which meant that trajectories with similar labels were not necessarily equivalent empirical constructs across groups.

We have corrected this issue in the revised manuscript. Trajectory clusters are now estimated within each baseline age group using the full pooled age-group-specific sample. After creating this common typology for each age group, we compare the sex and educational composition of the resulting shared trajectory types. This ensures that comparisons across women and men, and across educational groups, refer to common trajectory categories within each baseline age group.

This revised strategy is introduced in the Abstract and developed in the Materials and Methods section, especially in the subsections “Sequence analysis and trajectory classification” and “Analytical strategy”. The Results section then applies this strategy by presenting trajectory types estimated within pooled age-group samples and by comparing sex and educational composition within those shared types. The Discussion and Conclusion explicitly interpret this methodological change as a way to provide a comparable account of sex and educational differences in long-term functional ability trajectories.

We have also applied the same comparability logic to clarify the status of comparisons made across baseline age groups. Within each age group, the pooled estimation guarantees that sex and educational groups are compared within statistically shared trajectory categories. Across age groups, however, the grouping of the age-group-specific cluster solutions into three broad types (mostly non-declining, progressive-declining, and equivocal-declining) is an interpretive harmonization based on the shape, ordering, and timing of the detailed trajectories, rather than a single jointly estimated cross-age cluster solution. We now state this explicitly so that within-age comparisons (statistically grounded) and cross-age comparisons (descriptive) are not conflated. This clarification is reflected in the Results section, where the three broad types are introduced as a harmonization of the detailed age-group-specific trajectories, and in the Limitations subsection of the Discussion, where we note that comparisons across baseline age groups should be read as descriptive rather than as contrasts between strictly equivalent constructs.

3. The introduction lacks a clearly articulated research gap and does not sufficiently position the study within the existing literature on functional ability trajectories and sequence analysis methodology. Forever, the theoretical background and anchoring the present research in the current state of empirical results of other studies are somewhat weak. I should consider identifying, reviewing Relevant literature on the analyzed topic and strengthen this part of the paper.

Response: We have substantially rewritten the Introduction to address this concern. The revised Introduction now identifies two explicit research gaps. First, most evidence on long-term functional ability trajectories comes from the United States, Europe, and parts of Asia, while evidence from Latin America remains more limited. Second, the revised Introduction explains that comparisons across sex and educational groups require trajectory typologies constructed in a way that allows valid comparison across groups.

We also strengthened the theoretical and empirical framing. The revised Introduction discusses the shift from static disability measures toward longitudinal functional ability trajectories, the relevance of life-course and bridging approaches, previous international evidence on disability and functional ability trajectories, and prior Chilean evidence using the EPS. The revised version also clarifies why education is relevant as a marker of cumulative life-course resources.

These changes appear throughout the revised Introduction. The opening paragraphs now frame functional ability as a dynamic process and situate the study within longitudinal disability and functional ability research. The subsection “Research gaps” explicitly identifies the empirical and methodological gaps addressed by the study. The final paragraphs of the Introduction then clarify how the present study extends previous Chilean research and state the research question guiding the analysis.

4. Building methodological section, it should be provided the source of the data as the reference in the paper. Also, the reason the author decided to analyze should be explained: they thought we jar very old from 2004 to 2010.

Response: We revised the Materials and Methods section to provide a clearer description of the data source and the rationale for the analytic period and baseline age range. The revised manuscript now identifies the Encuesta de Protección Social (Social Protection Survey, EPS) as a nationally representative Chilean longitudinal survey and includes a reference to the official EPS data source.

We also explain why the 2004–2020 observation window was used. The period covers all the surveys available to date. This period provides six observation points with functional ability information and allows us to examine functional ability changes over 17 years, from midlife into older age. We further clarify why individuals aged 46–65 in 2004 were selected: this baseline age range allows the analysis to begin before most respondents reach older ages while still observing transitions into later life. Respondents were then divided into four five-year baseline age groups: 46–50, 51–55, 56–60, and 61–65.

These revisions are concentrated in the Materials and Methods section, particularly in the subsection “Data source and study design”. In that subsection, we now identify the EPS as the data source, cite the official EPS data reference, justify the 2004–2020 observation window, explain the selection of individuals aged 46–65 at baseline, and describe the rationale for analyzing four five-year baseline age groups separately.

5. By generating different cluster solutions within each stratified subgroup, the study creates group-specific trajectory types and then compares their prevalence as if they were equivalent constructs, which constitutes a methodological inconsistency.

Response: We agree with the Reviewer. This methodological inconsistency has been corrected. In the revised manuscript, we no longer generate sex-specific or education-specific cluster solutions. Instead, for each baseline age group, we estimate one pooled trajectory typology using the full age-group-specific sample. We then compare the sex and educational composition of the resulting shared trajectory types.

This revision changes the interpretation of the Results. The manuscript no longer compares the prevalence of non-equivalent clusters across separately stratified subgroup solutions. It now compares the distribution of sex and educational groups within common trajectory categories.

This correction is described in the Materials and Methods section, where we explain the pooled age-group-specific clustering strategy. The Results section then follows this revised design by presenting trajectory types defined before sex and education are compared. The subsections “Sex differences in trajectory types across age groups” and “Educational differences in trajectory types across age groups” report the composition of shared trajectory types by sex and education. Tables 2 and 3 support this analysis by reporting the sex and educational composition of main and detailed trajectory types within each age group. The Discussion explains why this revised strategy strengthens the validity of the comparative claims.

We would like to clarify one remaining aspect of comparability that follows from the Reviewer’s observation. The pooled estimation resolves the comparability problem for sex and educational groups within each baseline age group, because these groups are now compared within statistically equivalent trajectory categories. The grouping of the detailed, age-group-specific clusters into three broad types, however, is an interpretive harmonization rather than a jointly estimated typology spanning all age groups. We have made this distinction explicit to avoid implying that the underlying detailed clusters are statistically equivalent across age groups. Accordingly, the Results section now presents the three broad types as an interpretive grouping of the age-group-specific trajectories, intended to support cross-age description, and the Limitations subsection notes that comparisons across baseline age groups are descriptive in nature, while comparisons by sex and education within each age group rest on the shared, pooled trajectory categories.

6. When constructing the text, the ideas should be consolidated in Francis and paragraphs, not on short sentences and paragraph with 1 1/2 or two lines. Moreover, should be avoided structuring the text in very short bullet points, like on lines 208-212, 222-225, 231-234,

Response: We revised the manuscript’s writing style to address this comment. The previous Results section included short bullet-style descriptions of trajectory subtypes. In the revised manuscript, these descriptions have been rewritten as continuous prose. We also consolidated short paragraphs throughout the Introduction, Materials and Methods, Results, and Discussion sections to improve flow and readability.

This revision is especially visible in the Results section. The subsection “Functional ability trajectories across age groups” now describes the detailed trajectories in integrated paragraphs rather than short bullet points. The sex and education subsections also present group differences in narrative prose. The Discussion has been reorganized into interpretive paragraphs that synthesize the findings and reduce unnecessary repetition with the Results section.

7. Considering above consideration, the paper should undergo a major revision before being considered for publication.

Response: We agree that a major revision was necessary. The manuscript has been substantially revised in response to the Reviewer’s comments. The revised version includes a corrected analytical strategy, a clearer research gap, a stronger methodological explanation, updated figures and tables, a rewritten Results section based on pooled age-group-specific trajectory typologies, and a revised Discussion and Conclusion.

These changes affect the manuscript as a whole. The revision corrects the methodological inconsistency identified by the Reviewer, strengthens the Introduction and Methods, reorganizes the Results around comparable trajectory categories, and clarifies the interpretation of sex and educational differences in the Discussion and Conclusion. We believe these changes directly address the Reviewer’s concerns and substantially improve the manuscript’s methodological validity, clarity, and contribution.

Attachment Submitted filename: Response to Reviewers (2).docx

# Peer review: Decision Letter 1

**DOI:** 10.1371/journal.pone.0361826.r003

**Authors:** Oyelola A. Adegboye

28 Aug 2026

Dear Dr. Villalobos Dintrans,

Thank you for submitting your manuscript to PLOS One. After careful consideration, we feel that it has merit but does not fully meet PLOS One’s publication criteria as it currently stands. Therefore, we invite you to submit a revised version of the manuscript that addresses the points raised during the review process.

Please submit your revised manuscript by Oct 12 2026 11:59PM. If you will need more time than this to complete your revisions, please reply to this message or contact the journal office at plosone@plos.org. When you're ready to submit your revision, log on to https://www.editorialmanager.com/pone/ and select the 'Submissions Needing Revision' folder to locate your manuscript file.

- A letter that responds to each point raised by the academic editor and reviewer(s). You should upload this letter as a separate file labeled 'Response to Reviewers'.
- A marked-up copy of your manuscript that highlights changes made to the original version. You should upload this as a separate file labeled 'Revised Manuscript with Track Changes'.
- An unmarked version of your revised paper without tracked changes. You should upload this as a separate file labeled 'Manuscript'.

If you would like to make changes to your financial disclosure, please include your updated statement in your cover letter. Guidelines for resubmitting your figure files are available below the reviewer comments at the end of this letter.

If applicable, we recommend that you deposit your laboratory protocols in protocols.io to enhance the reproducibility of your results. Protocols.io assigns your protocol its own identifier (DOI) so that it can be cited independently in the future. For instructions see: https://journals.plos.org/plosone/s/submission-guidelines#loc-laboratory-protocols. Additionally, PLOS One offers an option for publishing peer-reviewed Lab Protocol articles, which describe protocols hosted on protocols.io. Read more information on sharing protocols at https://plos.org/protocols?utm_medium=editorial-email&utm_source=authorletters&utm_campaign=protocols.

As the corresponding author, your ORCID iD is verified in the submission system and will appear in the published article. PLOS supports the use of ORCID, and we encourage all coauthors to register for an ORCID iD and use it as well. Please encourage your coauthors to verify their ORCID iD within the submission system before final acceptance, as unverified ORCID iDs will not appear in the published article. *Only*  the individual author can complete the verification step; PLOS staff *cannot*  verify ORCID iDs on behalf of authors.

We look forward to receiving your revised manuscript.

Kind regards,

Oyelola A. Adegboye, PhD

Academic Editor

PLOS One

Journal Requirements:

If the reviewer comments include a recommendation to cite specific previously published works, please review and evaluate these publications to determine whether they are relevant and should be cited. There is no requirement to cite these works unless the editor has indicated otherwise.

Please review your reference list to ensure that it is complete and correct. If you have cited papers that have been retracted, please include the rationale for doing so in the manuscript text, or remove these references and replace them with relevant current references. Any changes to the reference list should be mentioned in the rebuttal letter that accompanies your revised manuscript. If you need to cite a retracted article, indicate the article’s retracted status in the References list and also include a citation and full reference for the retraction notice.

[Note: HTML markup is below. Please do not edit.]

Reviewers' comments:

Reviewer's Responses to Questions

**Comments to the Author**

Reviewer #2: (No Response)

**********

2. Is the manuscript technically sound, and do the data support the conclusions?

Reviewer #2: Yes

**********

3. Has the statistical analysis been performed appropriately and rigorously?

Reviewer #2: Yes

**********

4. Have the authors made all data underlying the findings in their manuscript fully available?

The PLOS Data policy

Reviewer #2: Yes

**********

5. Is the manuscript presented in an intelligible fashion and written in standard English?

Reviewer #2: Yes

**********

Reviewer #2: I thank the authors for their very interesting contribution, emphasising the dynamic nature of functional ability in Chile across time, and the potential heterogeneity of this across age, sex and education levels. I have the following comments on the different sections.

- Introduction:

o It is stated that previous research on high-income countries has shown that individual and social factors shape functional decline, but how this operates is not made explicit (i.e., what do we already know from these contexts?)

- Research gaps:

o It is argued that a main gap is that evidence so far comes mainly from the US, Europe and Asia, and that evidence from Latin America is scarce, even though the region faces rapid population ageing, high inequality and different institutional arrangements. I think the argument could be pushed a little further to say why these particularities make this research gap relevant (e.g., what should we expect to be different from/similar to what we already know from previous research?). This could also be further re-emphasised within the discussion or conclusion sections.

- Materials and methods:

o EPS waves selection: It is stated that the period 2004-2020 covers all EPS available waves. However, there is also a 2002 wave and a 2024 wave. I assume the former wasn’t used as it is not representative of the adult population but only of those affiliated to the pension system (or perhaps because of the lack of any relevant variable?) and the latter as it was probably not yet available when the analysis was undertaken, but it is worth mentioning whether this is the case.

o EPS waves limitations: Some additional issues with the EPS data might be acknowledged (and/or reflect on whether they might have any implications for the analysis). For instance, EPS 2012 data were published with some reservations due to low response rates during fieldwork and the institution responsible for the survey suggests caution when using them for statistical inference. In addition, fieldwork for the EPS 2020 wave had to be suspended during mid-March 2020 due to Covid-19 lockdown measures, resulting in a smaller sample size, and although the survey was continued from September to December of that year, it changed mode to a phone survey.

o Table 1 description: When describing Table 1, the acronym FA is used (in line 156). I assume it refers to functional ability, but I think it is not previously defined in the text. FA is also used in line 225 when describing the sequence analysis.

o Analytical sample: The analytical sample is composed of 2,681 individuals aged 45-64 in 2004 across the 6 EPS waves. Is this sample representative of the population in that age group? (i.e., how might the attrition of the survey be affecting this, given that the analyses may miss people who are only present in some of the waves?).

o Sampling weights: Does the analysis use any survey sampling weights? This should be stated, and the reason for the choice justified.

- Results and discussion:

o When presenting/discussing the findings of the sex and educational differences in trajectory types across age groups, it will be interesting to relate them to the respective prevalent differences in sex and educational categories by each group and whether or not it is consistent. For example, the mostly non-declining trajectories had a higher proportion of men in age groups 1, 3, and 4 and the progressive-declining trajectories had a higher proportion of women in those same age groups. Given that age groups 1 and 2 overall have more women than men and age groups 3 and 4 more men than women, the disaggregation by trajectory types may be “reverting” or “reinforcing” some of this aggregate composition. The same applies to the education findings discussion.

**********

what does this mean?). If published, this will include your full peer review and any attached files.

If you choose “no”, your identity will remain anonymous but your review may still be made public.

**Do you want your identity to be public for this peer review?**  For information about this choice, including consent withdrawal, please see our Privacy Policy

Reviewer #2: No

**********

[NOTE: If reviewer comments were submitted as an attachment file, they will be attached to this email and accessible via the submission site. Please log into your account, locate the manuscript record, and check for the action link "View Attachments". If this link does not appear, there are no attachment files.]

To ensure your figures meet our technical requirements, please review our figure guidelines: https://journals.plos.org/plosone/s/figures

You may also use PLOS’s free figure tool, NAAS, to help you prepare publication quality figures: https://journals.plos.org/plosone/s/figures#loc-tools-for-figure-preparation.

NAAS will assess whether your figures meet our technical requirements by comparing each figure against our figure specifications.

# Author comment: Author response to Decision Letter 2

**DOI:** 10.1371/journal.pone.0361826.r004

29 Sep 2026

Response Letter

Dear Academic Editor and Reviewer,

We sincerely thank you for the opportunity to revise and resubmit our manuscript, “Sex and Educational Inequalities Across Functional Ability Trajectories in Chile: A Sequence Analysis of a 17-Year National Panel”. We are particularly grateful to the Reviewer for a set of comments that were detailed, constructive, and closely informed by the data source we use. Several of them identified issues that we had not documented adequately and that we have now addressed throughout the manuscript.

The most important change concerns the interpretation of our central findings. Following the Reviewer’s eighth comment, we now reference the sex and educational composition of every trajectory type to the marginal composition of its baseline age group, both in Tables 2 and 3 and in the Results and Discussion. This has materially improved the analysis, as it allows us to distinguish compositional differences that reverse the aggregate distribution of an age group from those that reproduce it, and it revealed that one of our previous claims required qualification. The apparent concentration of respondents with primary education or less in equivocal-declining trajectories in the oldest age group (65.7%) in fact falls slightly below that age group’s marginal (67.0%), and all three trajectory types in that age group track the marginal educational distribution within 7.2 percentage points, which now provides a substantive account of why the chi-squared test is non-significant there.

Beyond this, we have made the following changes. The Introduction now specifies the mechanisms through which individual and social factors shape functional decline in high-income settings. The Research gaps subsection articulates why the Chilean configuration of rapid demographic aging, recent educational expansion, a historically deregulated labor market, and a segmented health and long-term care system leads to specific expectations about how functional trajectories unfold, and the Discussion returns to these expectations. The Materials and Methods section now documents the selection of EPS waves explicitly, including the reasons for excluding the 2002, 2012, and 2024 waves, and acknowledges the disruption of the 2020 fieldwork. We have clarified the representativeness of the analytic sample, stated and justified the decision not to apply survey weights, and removed an undefined acronym. Six new references have been added and the reference list has been renumbered accordingly.

Below we respond to each comment in turn, indicating where the corresponding changes appear.

Yours sincerely,

The authors.

REVIEWER

Comment 1. It is stated that previous research on high-income countries has shown that individual and social factors shape functional decline, but how this operates is not made explicit (i.e., what do we already know from these contexts?)

Response: We thank the Reviewer for this observation. The previous version listed the factors associated with functional decline without specifying how they operate. We have rewritten the third paragraph of the Introduction to describe the mechanisms documented in high-income settings, distinguishing sex-related, socioeconomic, and clinical pathways.

The revised paragraph specifies the configuration underlying sex differences, namely a higher burden of non-fatal disabling conditions, differences in muscle mass and strength, and lifetime differences in paid work and caregiving responsibilities, alongside longer survival among women. It then specifies the intermediate channels through which educational attainment operates, including exposure to physically demanding and hazardous occupations, health literacy and the capacity to navigate health systems, income and material conditions in later life, and health behaviors. It also clarifies the dual role of chronic conditions, multimorbidity, and sedentary behavior, which act both as precipitating events that trigger transitions into limitation and as constraints on the likelihood of recovering function once limitation has occurred, so that advantaged groups more frequently display recovery and less frequently remain in unstable states. We further note that these mechanisms accumulate across the life course, with early-life circumstances and adulthood employment histories shaping functional status decades later, and that the strength and direction of specific associations varies across age groups and cohorts.

Two references on sex differences in health and survival and one on childhood socioeconomic position and later-life functional limitations in Chile have been added to support these mechanisms.

Comment 2. It is argued that a main gap is that evidence so far comes mainly from the US, Europe and Asia, and that evidence from Latin America is scarce, even though the region faces rapid population ageing, high inequality and different institutional arrangements. I think the argument could be pushed a little further to say why these particularities make this research gap relevant (e.g., what should we expect to be different from/similar to what we already know from previous research?). This could also be further re-emphasised within the discussion or conclusion sections.

Response: We agree that the argument required further development. The previous version stated that the region differs from the settings where most evidence has been produced without specifying what those differences imply for the phenomenon under study. We have added a new paragraph to the Research Gaps subsection that articulates why the Chilean configuration matters and what it leads us to expect.

Specifically, we now discuss four features and their implications. The compressed pace of demographic aging means that successive cohorts reach later life with markedly different educational and occupational endowments. Rapid educational expansion produces steep cohort gradients in educational attainment, which entangles educational differences with cohort composition and shifts the social meaning of a given educational threshold across baseline age groups. A labor market characterized by weak employment protection and persistent informality concentrates cumulative occupational exposure among lower-educated workers, which plausibly produces steeper educational gradients than in settings with stronger labor regulation. A segmented health system and limited long-term care provision make recovery from an episode of limitation dependent on household resources, which suggests that socioeconomic differences will be expressed in the stability and reversibility of trajectories as much as in their onset. We also state which features we expect to resemble those documented elsewhere, namely the basic heterogeneity of functional pathways.

Following the Reviewer's suggestion, we return to this argument in the Discussion with a dedicated paragraph that assesses our findings against these expectations. First, we note that the concentration of women and of lower-educated respondents in equivocal-declining trajectories is consistent with recovery being conditional on household resources. Second, we observe that the strengthening of the educational gradient in progressive-declining trajectories with baseline age is consistent with cumulative occupational exposure. Finally, we argue that the heterogeneity of the trajectories themselves resembles that reported in higher-income settings, so that the distinctiveness of the Chilean case lies in how social groups are distributed across these pathways rather than in the existence of the pathways themselves. The Conclusion has been adjusted accordingly.

Comment 3. EPS waves selection: It is stated that the period 2004-2020 covers all EPS available waves. However, there is also a 2002 wave and a 2024 wave. I assume the former wasn’t used as it is not representative of the adult population but only of those affiliated to the pension system (or perhaps because of the lack of any relevant variable?) and the latter as it was probably not yet available when the analysis was undertaken, but it is worth mentioning whether this is the case.

Response: We thank the Reviewer for noting this, and we confirm that both assumptions are correct. The statement that the study used all available EPS waves was inaccurate and has been removed.

The revised Data source and study design subsection now lists all waves fielded by the EPS (2002, 2004, 2006, 2009, 2012, 2015, 2018, 2020, and 2024) and explains the selection of the analytic window. The 2002 wave was excluded for the two reasons the Reviewer anticipates, that is, questions on functional ability status were incorporated into the survey only from the 2004 version onwards, and the 2002 sample was restricted to individuals affiliated to the pension system, which limits its representativeness of the adult population. The 2024 wave had not been released at the time the analyses were conducted, and we now state that it will be incorporated into future extensions of this work. Additionally, the 2012 wave was also excluded, for reasons of data quality that we detail in our response to Comment 4 below.

Comment 4. EPS waves limitations: Some additional issues with the EPS data might be acknowledged (and/or reflect on whether they might have any implications for the analysis). For instance, EPS 2012 data were published with some reservations due to low response rates during fieldwork and the institution responsible for the survey suggests caution when using them for statistical inference. In addition, fieldwork for the EPS 2020 wave had to be suspended during mid-March 2020 due to Covid-19 lockdown measures, resulting in a smaller sample size, and although the survey was continued from September to December of that year, it changed mode to a phone survey.

Response: We are grateful for this observation, which led us to document two data-quality issues that the previous version did not acknowledge.

The revised Data source and study design subsection now includes a dedicated paragraph stating that the 2012 wave was published with reservations by the institution responsible for the survey, due to low response rates during fieldwork and an accompanying recommendation that these data be used with caution for statistical inference, and that it was therefore excluded from the analytic window. The same paragraph describes the disruption of the 2020 fieldwork, which began face-to-face in late 2019, was suspended in March 2020 following the introduction of nationwide lockdown measures, and resumed between September and December of that year by telephone, yielding a smaller achieved sample and a change in survey mode for part of the wave.

We have also added a new limitation (now the third) reflecting on the implications for our analysis. We acknowledge that mode effects on the self-report of difficulties in activities of daily living cannot be ruled out, and that the reduced achieved sample may have introduced additional selectivity at the endpoint of the observation window.

We note three features of our design that limit the leverage of this issue on our conclusions. First, trajectories are constructed from whole sequences of six observations, so that any single wave exerts bounded influence on cluster assignment. Second, the functional ability measure is a coarse hierarchical classification that is less sensitive to fine-grained reporting differences than a continuous score. And third, the sensitivity analysis based on the number of reported limitations reproduced the broad trajectory structure. We state that results concerning the final period should nonetheless be interpreted with this caveat in mind.

Comment 5. Table 1 description: When describing Table 1, the acronym FA is used (in line 156). I assume it refers to functional ability, but I think it is not previously defined in the text. FA is also used in line 225 when describing the sequence analysis.

Response: We thank the Reviewer for identifying this inconsistency. The acronym was indeed used without prior definition. Since it appeared only twice, we have replaced both occurrences with the full term “functional ability” rather than introducing an abbreviation, which improves readability.

The affected passages are the description of Table 1 in the Data source and study design subsection and the description of the cluster selection procedure in the Sequence analysis and trajectory classification subsection.

Comment 6. Analytical sample: The analytical sample is composed of 2,681 individuals aged 45-64 in 2004 across the 6 EPS waves. Is this sample representative of the population in that age group? (i.e., how might the attrition of the survey be affecting this, given that the analyses may miss people who are only present in some of the waves?).

Response: We thank the Reviewer for raising this point, which we have addressed in two ways.

First, we now state explicitly that the analytic sample is defined by observability of functional ability status across the six waves, and is therefore representative of respondents who remained in the panel rather than of the Chilean population aged 46–65 in 2004.

Second, we have expanded the discussion of Supplementary Table 1, which compares the analytic sample with the broader sample that includes individuals with missing observations. The differences are modest and consistent in direction across age groups. The analytic sample contains between 3.4 and 5.0 percentage points more respondents with primary education or less across the four age groups, a somewhat higher proportion of women in the three younger age groups, and a marginally lower baseline prevalence of no limitations in three of the four age groups. Retention therefore does not appear to have selected the most advantaged or the healthiest respondents, which is reassuring for a study concerned with functional decline. We also note that retaining individuals who died during the observation period, and treating death as a functional ability state, limits one of the principal sources of selective loss in long panels of aging populations.

The second limitation has been expanded accordingly, acknowledging that individuals lost to follow-up for reasons correlated with unobserved health deterioration cannot be recovered and that some selection bias cannot be ruled out.

Comment 7. Sampling weights: Does the analysis use any survey sampling weights? This should be stated, and the reason for the choice justified.

Response: We thank the Reviewer for this request. The analyses are unweighted, and the previous version neither stated nor justified this decision. We have now made it explicit in a new paragraph of the Analytical Strategy subsection and provided three reasons.

First, the construction of the typology rests on pairwise dissimilarities between sequences and on hierarchical clustering, procedures into which expansion factors cannot be incorporated in a straightforward manner at the stage where trajectory types are defined. Second, our substantive claims concern the sex and educational composition of shared trajectory categories within each baseline age group, a within-sample comparison for which weighting is less consequential than it would be for national prevalence estimation. Third, previous research using the same survey and a comparable trajectory design reported highly similar results with and without the expansion factor, which is consistent with an accurate sampling strategy and adequate fieldwork in the EPS.

We have also added a new limitation (now the fourth) stating that, because analyses are unweighted, the prevalence figures reported for each trajectory type describe the analytic sample and should not be read as population estimates, while noting that the compositional comparisons supporting our substantive claims are internal to each age group.

Comment 8. When presenting/discussing the findings of the sex and educational differences in trajectory types across age groups, it will be interesting to relate them to the respective prevalent differences in sex and educational categories by each group and whether or not it is consistent. For example, the mostly non-declining trajectories had a higher proportion of men in age groups 1, 3, and 4 and the progressive-declining trajectories had a higher proportion of women in those same age groups. Given that age groups 1 and 2 overall have more women than men and age groups 3 and 4 more men than women, the disaggregation by trajectory types may be “reverting” or “reinforcing” some of this aggregate composition. The same applies to the education findings discussion.

Response: We are grateful for this suggestion, which substantially improved the interpretation of our findings and led to the most extensive revision in this round. The previous version reported the composition of each trajectory type without anchoring it to the overall composition of the corresponding baseline age group, so that readers could not assess whether a given composition departed from or reproduced the aggregate distribution.

We have addressed this in four ways.

First, Tables 2 and 3 now include a first row reporting the marginal sex and educational distribution of each baseline age group, so that over- and under-representation can be read directly from the tables. The table notes explain this addition.

Second, the sex subsection now opens with an explicit statement of the marginals and of the convention we adopt, following the Reviewer’s own terms, that is, a departure is described as reversing the aggregate composition when the majority within a trajectory type differs from the majority in the age group, and as reinforcing it when the two coincide. Every composition is then expressed as a departure in percentage points. The male majority in mostly non-declining trajectories reverses the aggregate composition in age groups 1 and 2 (departures of 6.2 and 5.6 percentage points from female-majority marginals) and reinforces it in age groups 3 and 4 (4.5 and 8.6 percentage points). The female majority in equivocal-declining trajectories reinforces the aggregate composition in age groups 1 and 2 (10.2 and 8.0 percentage points) and reverses it in age group 4, where it constitutes the largest departure in the table at 18.4 percentage points. In age group 3 this type closely reproduces a nearly balanced marginal. Among progressive-declining trajectories, three of the four departures reverse the aggregate composition.

Third, the educational subsection follows the same logic. Mostly non-declining trajectories fall below the marginal proportion with primary education or less in all four age groups, by 4.2, 5.8, 4.7, and 3.1 percentage points. Progressive-declining trajectories move from 3.8 percentage points below the marginal in age group 1 to 3.9, 5.5, and 7.2 percentage points above it in age groups 2, 3, and 4, so that the educational gradient strengthens with baseline age relative to the composition of each age group and not only in absolute terms. Equivocal-declining trajectories exceed the marginal by 8.9, 5.8, and 10.7 percentage points in the three younger age groups.

A qualification of our earlier claim. Applying this framework revealed that one statement in the previous version required correction. We had described equivocal-declining trajectories as predominantly composed of lower-educated respondents in all four age groups, citing 65.7% in age group 4. Referenced to that age group’s marginal of 67.0%, this composition in fact falls 1.3 percentage points below the aggregate distribution and therefore does not indicate over-representation. We now state this explicitly, and we note that all three trajectory types in age group 4 fall within 7.2 percentage points of the marginal educational distribution, which provides a substantive interpretation of the non-significant chi-squared test in that age group. The Abstract has been amended so that the educational claim is restricted to the three younger age groups.

The Discussion now includes a dedicated paragraph drawing these threads together, and the Conclusion notes that aging research benefits from referencing trajectory composition to the aggregate composition of the population under study.

We thank again the Reviewer for giving us the opportunity to improve our study.

Attachment Submitted filename: Response_to_Reviewers_(2)_auresp_2.docx

# Peer review: Decision Letter 2

**DOI:** 10.1371/journal.pone.0361826.r005

**Authors:** Oyelola A. Adegboye

16 Oct 2026

Sex and Educational Inequalities Across Functional Ability Trajectories in Chile: A Sequence Analysis of a 17-Year National Panel

PONE-D-26-00167R2

Dear Dr. Villalobos Dintrans,

We’re pleased to inform you that your manuscript has been judged scientifically suitable for publication and will be formally accepted for publication once it meets all outstanding technical requirements.

Within one week, you’ll receive an e-mail detailing the required amendments. When these have been addressed, you’ll receive a formal acceptance letter and your manuscript will be scheduled for publication.

An invoice will be generated when your article is formally accepted. Please note, if your institution has a publishing partnership with PLOS and your article meets the relevant criteria, all or part of your publication costs will be covered. Please make sure your user information is up-to-date by logging into Editorial Manager at Editorial Manager® and clicking the ‘Update My Information' link at the top of the page. For questions related to billing, please contact billing support.

If your institution or institutions have a press office, please notify them about your upcoming paper to help maximize its impact. If they’ll be preparing press materials, please inform our press team as soon as possible -- no later than 48 hours after receiving the formal acceptance. Your manuscript will remain under strict press embargo until 2 pm Eastern Time on the date of publication. For more information, please contact onepress@plos.org.

Kind regards,

Oyelola A. Adegboye, PhD

Academic Editor

PLOS One

Additional Editor Comments (optional):

Reviewers' comments:

Reviewer's Responses to Questions

**Comments to the Author**

Reviewer #2: All comments have been addressed

**********

2. Is the manuscript technically sound, and do the data support the conclusions?

Reviewer #2: Yes

**********

3. Has the statistical analysis been performed appropriately and rigorously?

Reviewer #2: Yes

**********

4. Have the authors made all data underlying the findings in their manuscript fully available?

The PLOS Data policy

Reviewer #2: Yes

**********

5. Is the manuscript presented in an intelligible fashion and written in standard English?

Reviewer #2: Yes

**********

Reviewer #2: (No Response)

**********

what does this mean?). If published, this will include your full peer review and any attached files.

If you choose “no”, your identity will remain anonymous but your review may still be made public.

**Do you want your identity to be public for this peer review?**  For information about this choice, including consent withdrawal, please see our Privacy Policy

Reviewer #2: **Yes:**  Joaquín Mayorga Camus

**********

# Editor report: Acceptance letter

**DOI:** 10.1371/journal.pone.0361826.r006

**Authors:** Oyelola A. Adegboye

PONE-D-26-00167R2

PLOS One

Dear Dr. Dintrans,

I'm pleased to inform you that your manuscript has been deemed suitable for publication in PLOS One. Congratulations! Your manuscript is now being handed over to our production team.

At this stage, our production department will prepare your paper for publication. This includes ensuring the following:

* All references, tables, and figures are properly cited

* All relevant supporting information is included in the manuscript submission,

* There are no issues that prevent the paper from being properly typeset

You will receive further instructions from the production team, including instructions on how to review your proof when it is ready. Please keep in mind that we are working through a large volume of accepted articles, so please give us a few days to review your paper and let you know the next and final steps.

Lastly, if your institution or institutions have a press office, please let them know about your upcoming paper now to help maximize its impact. If they'll be preparing press materials, please inform our press team within the next 48 hours. Your manuscript will remain under strict press embargo until 2 pm Eastern Time on the date of publication. For more information, please contact onepress@plos.org.

You will receive an invoice from PLOS for your publication fee after your manuscript has reached the completed accept phase. If you receive an email requesting payment before acceptance or for any other service, this may be a phishing scheme. Learn how to identify phishing emails and protect your accounts at https://explore.plos.org/phishing.

If we can help with anything else, please email us at customercare@plos.org.

Thank you for submitting your work to PLOS One and supporting open access.

Kind regards,

PLOS One Editorial Office Staff

on behalf of

Assoc Prof Oyelola A. Adegboye

Academic Editor

PLOS One
